# Evolution of Sensory Receptors

**DOI:** 10.1146/annurev-cellbio-120123-112853

**Published:** 2024-09-21

**Authors:** Wendy A. Valencia-Montoya, Naomi E. Pierce, Nicholas W. Bellono

**Affiliations:** 1 Department of Organismic and Evolutionary Biology and Museum of Comparative Zoology, Harvard University, Cambridge, Massachusetts, USA; 2 Department of Molecular and Cellular Biology, Harvard University, Cambridge, Massachusetts, USA

**Keywords:** ion channels, chemoreceptors, thermoreceptors, mechanoreceptors, light receptors, sensory drive

## Abstract

Sensory receptors are at the interface between an organism and its environment and thus represent key sites for biological innovation. Here, we survey major sensory receptor families to uncover emerging evolutionary patterns. Receptors for touch, temperature, and light constitute part of the ancestral sensory toolkit of animals, often predating the evolution of multicellularity and the nervous system. In contrast, chemoreceptors exhibit a dynamic history of lineage-specific expansions and contractions correlated with the disparate complexity of chemical environments. A recurring theme includes independent transitions from neurotransmitter receptors to sensory receptors of diverse stimuli from the outside world. We then provide an overview of the evolutionary mechanisms underlying sensory receptor diversification and highlight examples where signatures of natural selection are used to identify novel sensory adaptations. Finally, we discuss sensory receptors as evolutionary hotspots driving reproductive isolation and speciation, thereby contributing to the stunning diversity of animals.

## 1. INTRODUCTION

Sensory systems serve as the primary interface with the outside world, allowing organisms to perceive environmental cues critical to their survival. Sensory system function is mediated by a host of molecular receptors that detect and transduce specific external energy, chemicals, or physical forces into biochemical events and neural activity to elicit behavior (Julius & Nathans 2012). Adaptations in sensory receptor function therefore play a crucial role in shaping the ability of different species to occupy specific ecological and behavioral niches. Furthermore, considering the clear link between genotype and phenotype, sensory receptors represent a tractable model for dissecting the molecular basis of adaptation (Baldwin & Ko 2020, Wray 2013). Thus, combining evolutionary and functional studies of sensory receptors across species provides fertile ground to advance our understanding of how ecological and physiological factors shape genetic variation.

Numerous recent studies have investigated the evolutionary dynamics of sensory receptors within various groups, such as vertebrates and insects, as well as among specific receptor families (Baldwin & Ko 2020, Churcher & Taylor 2011, Eyun et al. 2017, Feuda et al. 2012, Guo et al. 2022, Himmel & Cox 2020, Kadowaki 2015, Leung & Montell 2017, Nei et al. 2008, Ni 2021, Peng et al. 2015, Policarpo et al. 2024, Robertson 2019, Saina et al. 2015, Saito & Tominaga 2015, Sparks et al. 2018, Vidal et al. 2018, Wicher & Miazzi 2021). Yet, a systematic survey of diverse sensory receptor families, including all major animal groups, holds the potential to uncover general patterns in the functional evolution of genes. This is because identifying large-scale patterns requires integration across the phylogenetic diversity of animals. In addition, animals are an ideal model to characterize conserved evolutionary themes. First, key innovations in early animal history, such as the origin of multicellularity and the evolution of nervous systems, have spurred the emergence of intricate and highly integrated sensory systems. Second, unlike other multicellular organisms, animals face the challenges of active exploration and navigation and thus display a wide range of complex and remarkably coordinated behaviors. Finally, animals have adapted to an extraordinary diversity of niches, playing crucial roles in ecosystems as predators, prey, pollinators, seed dispersers, and nutrient recyclers. This range of adaptations has evolved in response to the same fundamental types of physical stimuli, such as light, chemicals, stretch, and temperature, among others, which impinge upon sensory receptors and are decoded by animal sensory systems across the diversity of life histories (Block 1992). Hence, a comprehensive comparative analysis could reveal patterns of both conservatism and innovation in the evolution of sensory receptors.

**Figure 1:**
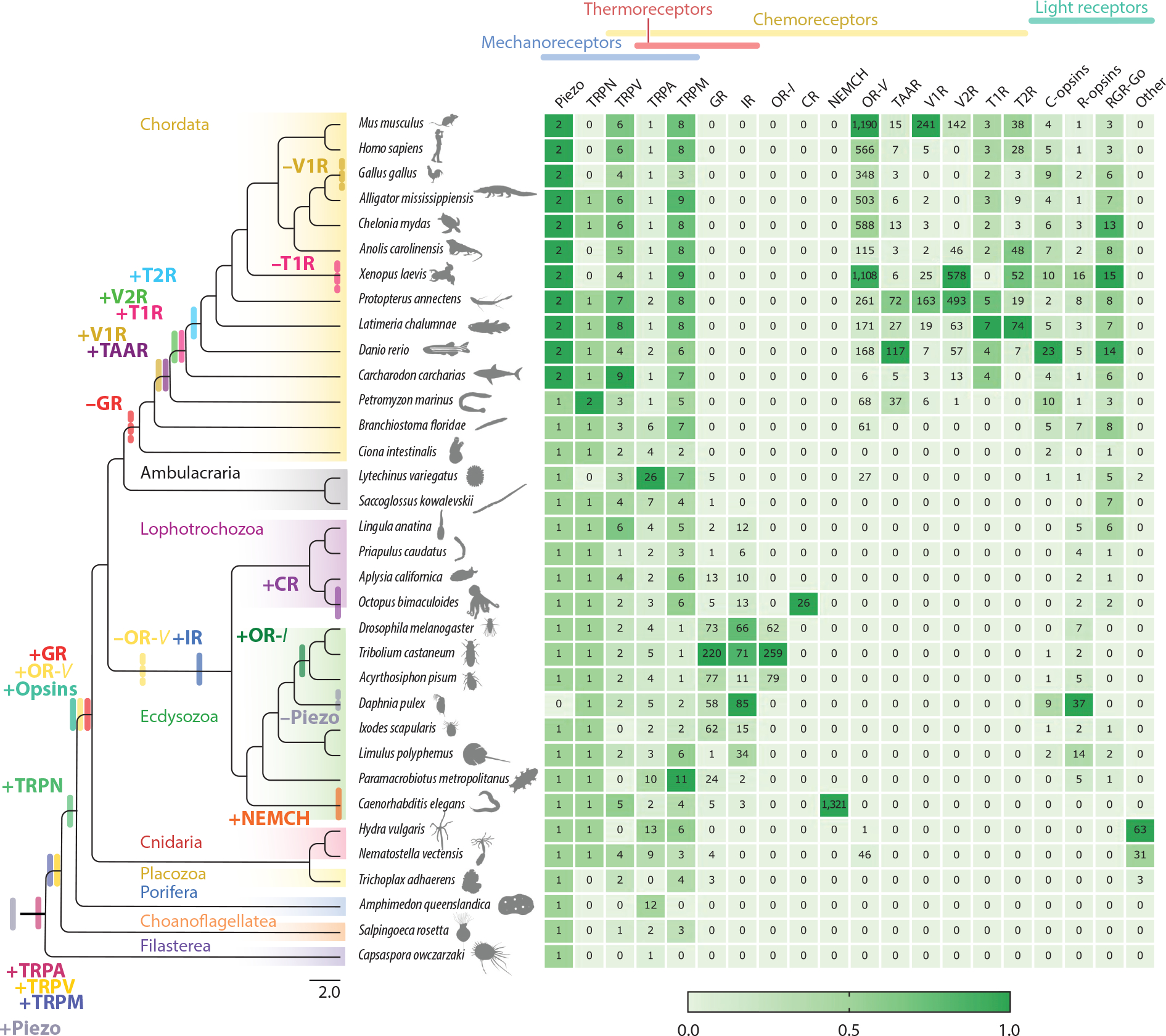
Evolution of major sensory receptor families. Sensory gene repertoires show lineage-specific expansions and losses, with chemoreceptors exhibiting the most dynamic patterns of evolution. The phylogeny of major metazoan clades, as well as the closest relatives of animals, the choanoflagellates and filastereans, is shown. Lines at the nodes indicate inferred gains, and dotted lines denote inferred losses of receptor families. Colors in the heat map represent the normalized number of genes across columns. The heat map values correspond to the number of retrieved receptors for each family and species. Sensory receptor families are classified as mechanoreceptors, thermoreceptors, chemoreceptors, and light receptors: Piezo; transient receptor potential (TRP) channels, including TRPM (melastatin), TRPV (vanilloid), TRPA (ankyrin), TRPP (polycystin or polycystic kidney disease), and TRPN (including *nompC*, or *no mechanorecaptor potential C*); gustatory receptor (GR); odorant receptor, mainly present in vertebrates (OR-*V*); ionotropic receptor (IR); chemotactile receptor (CR); odorant binding protein (OBP) and insect odorant receptor (OR-*I*); nematode chemosensory (NEMCH) receptor; trace amine-associated receptor (TAAR); vomeronasal receptor type 1 (V1R); vomeronasal receptor type 2 (V2R); taste receptor type 1 (T1R); taste receptor type 2 (T2R); ciliary opsins (C-opsins); rhabdomeric opsins (R-opsins); retinal G protein–coupled receptor (RGR-Go) opsins; and other (cnidopsins, placopsins, and echinopsins). Here, we used OR-*I* to distinguish insect ORs from the unrelated vertebrate odorant receptors, here called OR-*V*. Figure animal silhouettes adapted from images from https://www.phylopic.org/.

In this review, we present a brief overview of the evolutionary history of key sensory receptor families spanning all major clades of animals (here synonymous with metazoans) and closely related groups. We begin by mapping the gains and losses of these families throughout the animal phylogeny and discuss their repertoire size, as well as trends of lineage-specific expansions and contractions. Our estimates of gains, losses, and repertoire sizes are based on literature (Baldwin & Ko 2020, Churcher & Taylor 2011, Eyun et al. 2017, Feuda et al. 2012, Guo et al. 2022, Himmel & Cox 2020, Kadowaki 2015, Leung & Montell 2017, Nei et al. 2008, Ni 2021, Peng et al. 2015, Policarpo et al. 2024, Robertson 2019, Saina et al. 2015, Saito & Tominaga 2015, Sparks et al. 2018, Vidal et al. 2018, Wicher & Miazzi 2021) as well as the mining of annotated genomes from exemplar taxa (Figure 1) (see the Supplemental Appendix for details on receptor mining). Importantly, as placeholders for large clades in the phylogeny, exemplars provide a partial picture of the variation between clades. We then focus on the evolutionary mechanisms underlying the emergence of genetic variation and molecular adaptations in these receptor families. We devote particular attention to functional investigations of sensory receptors across levels of biological organization, from molecular and cellular properties to the broader context of organismal life history and ecology. Finally, we review recent work detecting natural selection and describing how sensory receptors represent hotspots in the evolution of reproductive isolation, ultimately catalyzing adaptive radiations and contributing to animal biodiversity.

## 2. EVOLUTIONARY HISTORY OF MAJOR SENSORY RECEPTOR FAMILIES IN ANIMALS

The oldest known animals existed ~600 million years ago during the Ediacaran period at the close of the Precambrian era (Bobrovskiy et al. 2018, Chen et al. 2019). Diverse lineages made their first appearance in the fossil record as marine forms during the Cambrian explosion around 541 million years ago (Dunn et al. 2014). Five major phyla arose during this period and survived to the present time: poriferans (sponges), ctenophores (comb jellies), placozoans (microscopic flat animals), cnidarians (anemones, jellyfish, and hydra), and bilaterians (including chordates, mollusks, arthropods, and a variety of worms) (Schultz et al. 2023). These extant groups comprise the animal kingdom, or Metazoa, which is sister to unicellular protists such as Choanoflagellata and Filasterea (Schultz et al. 2023). Whether sponges or ctenophores are a sister to the remaining metazoans, the common feature shared by all animals is multicellularity. Indeed, multicellularity is a pivotal trait for the evolution of sensory systems because it enabled the development of complex body plans with specialized cell types, organ morphologies, and receptors.

Beyond multicellularity, nervous systems represent another hallmark of animal evolution. Yet not all animals have nervous systems. Indeed, many groups of sensory receptors that are widespread in animals predate the evolution of nervous systems. Sponges lack neurons but express molecular components associated with presynaptic function (Musser et al. 2021). Similarly, placozoans are devoid of nervous systems, but recent evidence suggests they possess neuron-like cells (Najle et al. 2023). Thus, ancestral sensory receptors likely expanded in concert with the evolution of new cell types, sensory organs, and the elaboration of the nervous system. Brains evolved in bilaterally symmetrical animals, Bilateria, a group roughly composed of Protostomia and Deuterostomia (Martín-Durán & Hejnol 2021). Protostomes encompass diverse groups of invertebrates, such as the Ecdysozoa (which notably grow by molting and include arthropods and nematodes) and the Lophotrochozoa (a major spiralia clade encompassing mollusks and annelids) (Dunn et al. 2014). In contrast, deuterostomes include the ambulacrarians (such as echinoderms and hemichordates) and their sister group, the chordates (including vertebrates) (Dunn et al. 2014). Nervous systems across these distinct groups amplify and integrate signals perceived by sensory receptors to orchestrate appropriate behavioral responses. In turn, the neural coordination of different sensory modalities led to the evolution of more complex sensory systems and diverse sensory receptor repertoires.

The evolution of multicellularity and nervous systems is thus key to understanding the striking diversity of sensory modalities. Collectively, animals have evolved to monitor just about everything, including light, chemicals, sound, pressure, body position, heat, acceleration, gravity, electrical and magnetic fields, and even the passage of time (Block 1992). Nonetheless, the perception of many of these stimuli is restricted to specific clades and requires highly specialized receptor cells and organs. In this section, we review broad families of mechanoreceptors, thermoreceptors, chemoreceptors, and light receptors. We focus on these sensory modalities because they are widespread in all animal groups, allowing us to infer general patterns and uncover mechanisms by which sensory receptor families originate and change over time.

### 2.1. Transient Receptor Potential Channels Are Part of the Ancestral Sensory Toolkit of Animals

**Figure 2:**
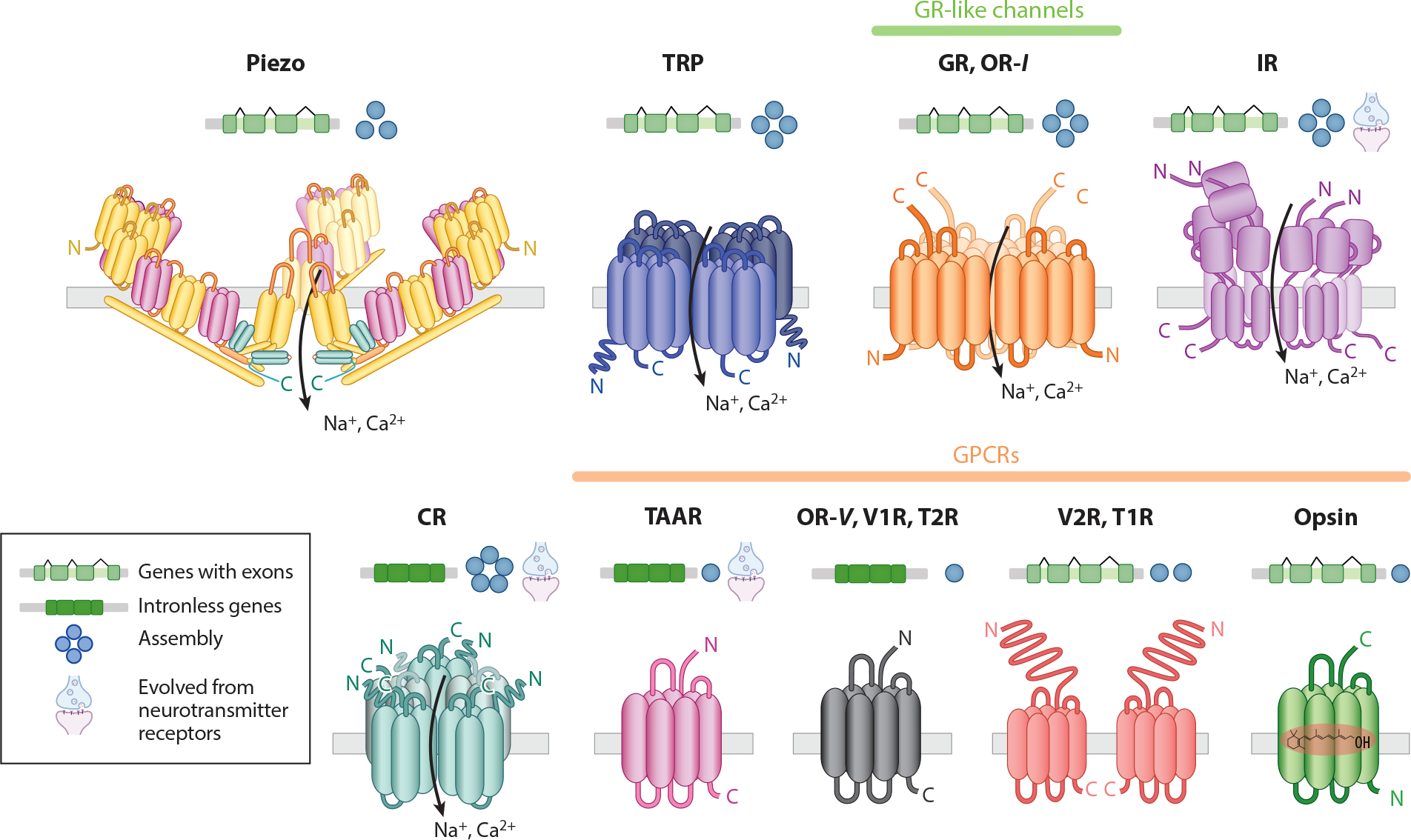
Genomic architecture, protein structure, and membrane topology of sensory receptors, showing patterns of assembly. Exon-intron structures (*green*) of sensory receptor families hint at the duplication mechanism underlying their evolution. The exon-intron architectures and assemblies were inferred from the majority of the known cases within the gene families. Distinct families across vertebrates and invertebrates diverged from ancestral neurotransmitter receptors, including IRs, CRs, and TAARs. Piezo channels, TRP channels, GRs, ORs-*I*, IRs, and CRs are nonselective cation channels. GPCRs transduce information through multicomponent second messenger–based signaling pathways. Abbreviations: CRs, chemotactile receptors; GPCRs, G protein–coupled receptors; GRs, gustatory receptors; IRs, ionotropic receptors; ORs-*I*, insect olfactory receptors; OR-*V*, vertebrate odorant receptor; T1R, taste receptor type 1; T2R, taste receptor type 2; TAARs, trace amine-associated receptors; TRP, transient receptor potential; V1R, vomeronasal receptor type 1; V2R, vomeronasal receptor type 2.

Transient receptor potential (TRP) channels are a large superfamily of receptors present in all animals that have been examined (Figures 1 and 2). TRP channels are activated by a diverse array of stimuli, including chemical, thermal, and mechanical signals, and function in numerous systems as signal transducers (Himmel & Cox 2020). TRP channels are widely expressed in eukaryotes but have not been reported in Archaea or Bacteria, suggesting they evolved early in eukaryote evolution (Cai & Clapham 2012, Himmel & Cox 2020, Himmel et al. 2020, Venkatachalam & Montell 2007). TRP receptors are divided into two major clades that diverged before plants and animals split. Group 1 likely predates the Cnidaria-Bilateria split (earlier than 750 Ma) and includes the TRPA (ankyrin), TRPM (melastatin), TRPN (including *nompC*, or *no mechanoreceptor potential C*), TRPC (canonical), TRPS (soromelastatin), TRPV (vanilloid), and TRPVL (vanilloid-like) subtypes. Since choanoflagellates have at least TRPM, TRPC, TRPV, and TRPA, these families also likely predate the emergence of animals (Himmel & Cox 2020). Contrastingly, group 2 split into TRPP (polycystin or polycystic kidney disease receptors) and TRPML (mucolipin) early in eukaryote evolution, as at least animals, alveolates, and other eukaryotes express genes from both subfamilies (Himmel & Cox 2020). The ancient nature of TRP channels, their myriad sensory functions, and conserved features across animal diversity provide hints that this receptor family may have functioned as an ancestral sensory toolkit of animals.

TRPV (see gene groups in Figure 1) channels underwent remarkable functional diversification during animal evolution (Figures 1 and 2). They are reported to detect stimuli ranging from temperature, chemical, and osmotic signals in vertebrates, to chemical and mechanical sensors in nematodes, and hygro- and mechanoreceptors in insects (Caterina et al. 1997, Himmel & Cox 2020, Rhyu et al. 2021). Placozoans and cnidarians have both inactive and nanchung TRPV subfamilies (Himmel & Cox 2020, Peng et al. 2015). Notably, choanoflagellate TRPV channels cluster more closely to mammalian TRPV than to inactive and nanchung, indicating that the TRPV-V subfamily was lost in the early protostome ancestor, whereas nanchung and inactive were lost in deuterostomes (Himmel & Cox 2020, Peng et al. 2015).

TRPN (*nompC*) channels function primarily as mechanoreceptors (Jin et al. 2017, van Giesen et al. 2020, Walker et al. 2000, Weir et al. 2020). They seem to be missing from placozoans, comb jellies, and choanoflagellates (Figure 1), suggesting that TRPN channels evolved in an early cnidarian-bilaterian ancestor after animals emerged (Peng et al. 2015, Schüler et al. 2015). Indeed, *nompC* mechanosensory function has been demonstrated in cnidarian stinging cells, suggesting nonneuronal origins (He et al. 2023, Weir et al. 2020).

TRPA channels are polymodal receptors that respond to a variety of stimuli in neurons and nonneuronal cell types. In different animals, they are gated by direct or indirect heat or cold, mechanical stimuli, hypoxia, electrophilic chemicals, reactive oxygen species, and endogenous molecules associated with tissue damage, among others (Himmel & Cox 2020). TRPA channels can be divided into TRPA1, TRPA1-like, and AsTRPA1 (arthropod TRPA channels or basal) (Himmel & Cox 2020). Channels in the TRPA1-like subfamily have not been functionally characterized but are found in cnidarians, ecdysozoans, and lophotrochozoans. Most arthropod-specific TRPA channels participate in high-temperature sensing, whereas *water witch* facilitates sound detection and hygrosensation (Lee et al. 2005, Liénard et al. 2024, Liu et al. 2007, Matsuura et al. 2009, Tracey et al. 2003). Unlike chemical sensitivity, the structural basis of temperature sensation is not well understood for TRPA proteins, although some evidence suggests that thermosensitivity is linked to highly conserved N-terminal ankyrin repeats (Cordero-Morales et al. 2011, Zhang et al. 2022).

TRPM channels are involved in temperature sensation, chemoreception, and mechanosensation (Huang et al. 2020). *TRPM8* is a well-characterized sensor for cold and menthol in vertebrates and insects (Himmel et al. 2019, McKemy et al. 2002). Choanoflagellates are unicellular and lack neurons, but they express TRPM channels, such as *TRPM2*, which shows enzymatic activity. In cnidarians, TRPM channels are widely expressed, including in neurons. Since TRPMs in both cnidarians and choanoflagellates can function as both Mg^2+^ channels and protein kinases, this might be their ancestral function (Lü & Du 2020, Tóth et al. 2020). This suggests that a sensory role of ancient nonneuronal TRP channels might have evolved from enzymatic activities and eventually been coopted by neurons for sensory integration.

In summary, TRP channels are fundamental components of a variety of sensory systems, predating the evolution of neurons. Interestingly, although TRP channels lack a canonical voltage-sensing domain, they are part of the large class of voltage-gated ion channels that includes voltage-gated K+, Na+, and Ca^2+^ channels (Arendt 2020). All of these voltage-gated ion channels are critical to signal transduction in neurons (Arendt 2020). Hence, given the ancient nature of TRP channels, their structural architecture, and their expression patterns, their evolution might contribute to understanding the early evolution of nervous systems. This is partly because sensory functions in TRP channels likely predate the emergence of the nervous system, which is often credited as the basis for animal behavior (Himmel & Cox 2020). Indeed, all TRP families appear in ancient nodes of the animal phylogeny and have been conserved throughout animal evolution (Figure 1). Therefore, TRP channels might represent the polymodal sensory receptor toolkit that endowed early animals with the capacity to respond to a wide repertoire of stimuli essential to the evolution of complex behaviors and physiology.

### 2.2. Mechanoreceptors Are Highly Conserved Over Evolutionary Time

Animals exploit subtle variations in both external and internal forces for navigation and communication. Mechanoreceptors transduce these forces to detect sound for hearing, body position for proprioception, and tactile sensation for touch (Katta et al. 2015). At least four major classes of membrane proteins have been linked to mechanotransduction in animals: TRP channels, degenerin/epithelial Na channels (DEG/EnaC), Piezo proteins, and transmembrane channel-like (TMC) proteins (Katta et al. 2015). Here, we focus on Piezo and TRP channels because they are widespread across animals (Figure 1) and have been robustly studied in heterologous systems that recapitulate the mechanically induced activation observed from native cells. Notably, these mechanoreceptor ion channels are remarkably conserved across animal groups.

Piezo proteins are mechanically activated ion channels with 24–40 transmembrane domains, making them among the largest known proteins (Figure 2) (Coste et al. 2010, Saotome et al. 2018). From an evolutionary perspective, Piezos are quite mysterious as they exhibit no homology to any other known ion channels (Katta et al. 2015). Nonetheless, they are reported among animals, plants, and single-cell eukaryotes, but are largely absent from bacteria and archaea, indicating they likely evolved in the common eukaryote ancestor, predating the evolution of animals (Katta et al. 2015). Conversely, TRPN is restricted to Eumetazoa (Cnidaria plus Bilateria) (Figure 1). Although Piezo and TRPN channels appeared in early animals, they have remained strikingly unchanged over the course of evolution, both in number of copies and in sequence identity. Piezo seems to have been duplicated just once at the base of the vertebrates, as they carry two copies, *Piezo1* and *Piezo2*, while invertebrates and tunicates only have *Piezo1* (Figure 1). Similarly, despite the occurrence of several independent whole-genome duplications across bilaterian taxa, only a single TRPN is predicted in bilaterian genomes (Figure 1) (Himmel & Cox 2020, Schüler et al. 2015). Since both Piezo and TRPN channels are highly conserved, mechanotransduction in animals seems to be mediated by a small repertoire of genes that play critical functions across physiological systems, whereby functional diversification might be driven by the diversity of cell types, expression patterns, auxiliary subunits, and organ morphology.

### 2.3. Chemoreceptors Exhibit a Dynamic History of Evolution, Including Numerous Gains and Losses

In stark contrast to other sensory modalities, chemoreceptors exhibit a dynamic evolutionary history. Chemoreceptors physically bind distinct chemicals that are waterborne, volatile, or encountered by contact. Indeed, the chemical world of animals is composed of a virtually infinite number of molecules varying in physicochemical properties and structure (Del Mármol et al. 2021). How do animals contend with the challenge of sensing the complex chemical world? One solution is having many chemoreceptors for many molecules. In fact, the number of chemoreceptors is several orders of magnitude greater than those used in other sensory modalities, and they constitute the largest known of any gene families (Figure 1). In addition to large repertoires, recent protein structural analyses suggest that promiscuity in the ligand-binding site of chemoreceptors could explain a broad coding logic for a vast diversity of chemical cues (Allard et al. 2023c, Billesbølle et al. 2023, Del Mármol et al. 2021, Kang et al. 2023, Ma et al. 2024). Therefore, both the diversity and promiscuousness of chemoreceptors underlie their critical role in mediating a wide range of behavioral responses such as feeding, hunting, mating, and avoiding predators.

#### 2.3.1. Gustatory receptors and insect odorant receptors.

Gustatory receptors (GRs) form ion channels activated by diverse chemicals, including sugar, bitter compounds, caffeine, salt, hydrocarbons, and amino acids (Bray & Amrein 2003, Delventhal & Carlson 2016, Fujii et al. 2015, Miyamoto et al. 2012, Shim et al. 2015). GRs also contribute to the sensation of CO_2_ and temperature (Jones et al. 2007, Ni et al. 2013), and given their diversity, additional functions likely await identification. GRs were first described in insects, but homologs have been found widely in protostomes, cnidarians, and placozoans, suggesting GRs predate the Cnidaria-Bilateria split and were secondarily lost in chordates (Figure 1) (Eyun et al. 2017). More recently, leveraging structure-based discovery of distant homologs, Himmel et al. (2023) identified GR-like receptors [including animal GRs and insect odorant receptors (ORs-*I*)] as part of an ancient and cryptic superfamily of 7-transmembrane domain ion channels (7TMICs) (Figure 2). Notably, while GRs are missing from chordates, they have undergone massive diversifications in invertebrates, particularly in insects (Figure 1). Indeed, odorant receptors (ORs-*I*) evolved from GRs concurrent with the terrestrial evolution of insects (ORs-*I* to distinguish them from the unrelated G protein–coupled receptors mostly found in vertebrates, ORs-*V*) (Benton et al. 2020, Brand et al. 2018, Missbach et al. 2014). ORs-*I* respond to a wide range of volatiles, including terpenes, aldehydes, alcohols, aromatic compounds, sex pheromones, and atmospheric gases, among others (De Bruyne et al. 2010, Del Mármol et al. 2021, Hallem & Carlson 2006, Hallem et al. 2004, Robertson 2019, Robertson et al. 2003, Sparks et al. 2018). Recent cryo-electron microscopy (cryo-EM) structures of GRs (Ma et al. 2024) and ORs-*I* (Butterwick et al. 2018, Del Mármol et al. 2021) show that they form tetrameric cation channels. Interestingly, although ORs-*I* can assemble as homomers, the most predominant system in insects is made from heteromeric channels composed of the ubiquitous and highly conserved *Orco* coreceptor and an OR tuning receptor that responds to specific ligands (Benton et al. 2006, Larsson et al. 2004).

#### 2.3.2. Olfactory receptors (ORs-*V*) and vomeronasal receptors.

The majority of chemosensory genes in chordates belong to the rhodopsin superfamily or class A G protein–coupled receptors (GPCRs) (Figure 2) (Nei et al. 2008, Nordström et al. 2011). ORs-*V* form the largest family of GPCRs (Nei et al. 2008). ORs-*V* are present in cnidarians and deuterostomes, implying that they evolved early in a Cnidaria-Bilateria ancestor and were subsequently lost in protostomes (Baldwin & Ko 2020, Churcher & Taylor 2011). ORs-*V* are primarily expressed in the olfactory epithelium but can function in other organs (Bellono et al. 2017, Pluznick et al. 2013). ORs are both broadly and narrowly tuned, responding to volatiles such as aldehydes, terpenes, thiols, ketones, alcohols, fatty acids, and aromatic compounds (Adipietro et al. 2012, Baldwin & Ko 2020, Fleischer et al. 2009, Jiang & Matsunami 2015, Nei et al. 2008). Pheromones, by contrast, are detected mainly by vomeronasal receptors (VRs) (Baldwin & Ko 2020, Dulac & Axel 1995). Interestingly, the two major families of VRs, V1Rs and V2Rs, originated before the emergence of a morphologically distinct vomeronasal organ (VNO) (Figure 1) (Baldwin & Ko 2020). V1Rs are older than V2Rs, and respond to sulfate steroids, volatile pheromones in urine, bile acids, and putative reproductive hormones (Doyle & Meeks 2018, Haga-Yamanaka et al. 2014, Meeks et al. 2010). Conversely, V2Rs first appeared in cartilaginous fish and, consistent with an aquatic environment, are activated by different soluble peptide pheromones and major histocompatibility complex peptides (Baldwin & Ko 2020, Karn & Laukaitis 2012, Laukaitis et al. 2008).

#### 2.3.3. Taste receptors: types 1 and 2.

Similar to V1Rs and V2Rs, the taste receptors T1R and T2R appeared at different nodes in the animal phylogeny (Figure 1) (Baldwin & Ko 2020). T1R receptors first evolved in cartilaginous fish, such as sharks. T1R repertoires are small and conserved, and although the bony ancestor vertebrate likely had nine copies, many tetrapod species only have three T1R genes (*T1R1*, *T1R2*, and *T1R3*) (Figures 1 and 2) that are broadly tuned to diverse tastants (Nishihara et al. 2024). Heterodimers of these genes bind sugar, amino acids, and certain nucleotides, and underlie sweet (*T1R2*/*T1R3*) (Nelson et al. 2001) and umami tastes (*T1R1*/*T1R3*) (Nelson et al. 2002). Taste receptors are mostly expressed in oral taste buds, but non-canonical expression has been demonstrated in diverse systems. For instance, catfish have taste buds covering their bodies and have been described as swimming tongues (Caprio et al. 1993). Similarly, sea robins are fish with leg-like appendages that express taste receptors in their epithelial cells to locate buried prey (Allard et al. 2023b). *T1R2* has been repeatedly pseudogenized in carnivore species (Policarpo et al. 2024), whereas hummingbirds and songbirds have convergently regained sweet perception by mutations in the ancestral umami receptor following the loss of *T1R2* early in bird evolution (Baldwin et al. 2014, Toda et al. 2021). By contrast, T2Rs, or bitter receptors, first appeared in bony vertebrates and show great variation in repertoire size (Figure 1).

In summary, the number of chemosensory receptor genes and families varies extensively among animals. Is the repertoire size correlated with ecological parameters or tuning breadth? Although functional studies remain rare, repertoire size appears to relate to tuning breadth and the complexity of a given species’ chemical ecology. For instance, in vertebrates, species with fewer chemosensory genes have broadly tuned receptors, while species with a higher number of genes show more narrowly tuned receptors (Baldwin & Ko 2020). In insects, generalist species tasting a wide variety of chemically defended plants show major expansions of divergent GRs (Robertson 2019). Furthermore, OR family expansions in ants and bees have been linked to the evolution of chemically based social communication (McKenzie et al. 2016, Robertson & Wanner 2006, Smith et al. 2011, Zhou et al. 2012). Another notable case of a lineage-specific expansion is the evolution of chemoreceptors with a limited phylogenetic distribution, such as the nematode chemosensory receptor (NEMCH) family, also from the rhodopsin superfamily (Nordström et al. 2011, Vidal et al. 2018). The genome of *Caenorhabditis elegans* encodes over 1,300 NEMCH genes, and functional studies have linked a subset of these receptors to the sensation of environmental and pheromonal cues (Vidal et al. 2018). Finally, a dramatic case of novel lineage-specific expansion and functional gain is the repeated evolution of chemoreceptors from ancestral neurotransmitter receptors in protostomes, cephalopods, and vertebrates, which we discuss below.

### 2.4. Independent Evolutionary Transitions of Chemoreceptors from Neurotransmitter Receptors

The emergence of neurotransmitter systems was likely triggered by the diversification of multicellular animals and the requirement for specialized communication among early nervous systems. These systems probably arose by independently coopting broadly tuned chemoreceptors into receptors narrowly tuned to specific neurotransmitters. More recently, we observed repeated evolutionary transitions from neurotransmitter receptors to peripheral chemoreceptors across ancient and derived nodes of the animal phylogeny (Figures 1 and 2). Examples include ionotropic receptors (IRs) evolving from ionotropic glutamate receptors (iGluRs) in protostomes (Benton et al. 2009, Croset et al. 2010) and trace amine-associated receptors (TAARs) from serotonergic receptors [5-hydroxytryptamine (5-HT)] in vertebrates (Dieris et al. 2021, Liberles & Buck 2006). The most recently described family of chemoreceptors, chemotactile receptors (CRs) in octopus and squid, diverged from ancestral nicotinic acetylcholine receptors (nAChRs) (Allard et al. 2023c, Kang et al. 2023, van Giesen et al. 2020). Like nAChRs, CRs form homopentameric and heteropentameric ion channels, but instead of binding acetylcholine, they are activated by hydrophobic molecules for contact-dependent aquatic chemosensation (Allard et al. 2023c, Kang et al. 2023, van Giesen et al. 2020). In octopuses, CRs have undergone significant expansions relative to other cephalopods, consistent with their elaborate chemotactile exploration of the seafloor (Allard et al. 2023a, c; Kang et al. 2023). For example, while the *Octopus bimaculoides* genome has up to 26 CRs, the *Doryteuthis pealeii* (squid) genome only encodes 6 (Kang et al. 2023).

Although the structure of an IR has not been determined, IRs are predicted to form ion channels similar to other iGluRs (Figures 1 and 2) (Ni 2021). However, in contrast to their ancestors, IRs are implicated in a remarkable diversity of sensory roles, including olfaction, gustation, hygrosensation, and thermosensation (Benton et al. 2009, Greppi et al. 2020, Morita et al. 2023, Ni 2021). IRs date to at least the emergence of protostomes, as they are present in arthropods, nematodes, and mollusks (Figure 1) (Croset et al. 2010, Eyun et al. 2017). Unlike CRs and IRs, which form ion channels, TAARs are GPCRs. TAARs evolved from 5-HT receptors and primarily specialize in detecting ecologically relevant amine compounds from animal body fluids (Guo et al. 2022, 2023). TAARs likely first appeared in the ancestor of vertebrates and are divided into TAARs and TAAR-like receptors, which include the TARL and the sea-lamprey-specific TARLL subgroups (Guo et al. 2022).

Although CRs, IRs, and TAARs represent striking cases of parallel evolutionary outcomes, all these transitions are quite ancient, occurring between 300 and 700 million years ago (Figure 1). Thus, more recent chemoreceptor families, such as formyl peptide receptors (FPRs), can offer clues about these evolutionary transitions. FPRs initially functioned as receptors for pathogens in the rodent immune system, but they have evolved into environmental sensors housed in the vomeronasal organ (VNO). Strikingly, Dietschi et al. (2017) found that through exon shuffling, an FPR immune receptor coopted a promoter of a VR, hijacking its expression pattern. Therefore, changes in regulatory elements seem sufficient to explain the switch of FPRs from detecting pathogens inside the organism to sensing the outside world (Dietschi et al. 2017). The initial transition from neurotransmitters might similarly involve changes in expression patterns, since the evolution of regulatory elements might be under relaxed selection compared to drastic functional modifications. Indeed, the basic function of neurotransmitters and chemoreceptors is conserved, as they both initiate cellular activity in response to binding an extracellular ligand, whether from a presynaptic partner or the external environment. Unraveling similar or distinct genomic mechanisms underlying the evolution of CRs, IRs, and TAARs from neurotransmitter receptors represents an exciting avenue for research in evolutionary sensory biology.

### 2.5. Receptors for Temperature and Light Are Related to Chemoreceptors

Chemosensation is one of the most ancient senses, and receptors involved in other sensory modalities are often nested within families of chemosensory genes. Notably, thermosensory proteins in animals have independently evolved many times, including in the TRP, IR, and GR families, whereby chemosensation is likely to be ancestral (Figure 1) (Caterina et al. 1997, Greppi et al. 2020, McKemy et al. 2002, Morita et al. 2023, Ni et al. 2013). Indeed, thermosensory TRP channels mediate a host of thermosensory responses, while maintaining conserved chemical sensitivity (Himmel & Cox 2020). For example, capsaicin, electrophiles, and menthol are agonists of *TRPV1*, *TRPA1*, and *TRPM8*, respectively, across diverse bilaterians (Himmel & Cox 2020). Similarly, *GR28b(D)*, a thermosensitive channel in *Drosophila*, is part of the large and ancient family of gustatory receptors (GRs) (Ni et al. 2013). More recently, IR genes, such as *IR21a*, *IR40*, *IR93a*, and *IR25a*, were shown to be necessary for responses to cooling in flies and mosquitoes (Greppi et al. 2020, Morita et al. 2023). Interestingly, temperature sensing in bacteria is also coupled to chemoreception. The bacterial *Tar* and *Tsr* proteins are both the major chemoreceptors and thermoreceptors used to navigate thermal and chemical gradients (Sengupta & Garrity 2013). Thus, these unrelated cases underscore the relationship between chemo- and thermosensory modalities across the tree of life and can offer clues into the molecular mechanisms of temperature sensing, which remain poorly understood.

Unlike diverse thermoreceptors, opsins represent a single gene family that mediates visual transduction across animals. Nonetheless, opsins are also derived from ancestral chemoreceptors, as they are part of the superfamily of rhodopsin-like GPCRs, which, in addition to opsins, includes hormone, neuropeptide, neurotransmitter, and olfactory receptors (Nordström et al. 2011). Yet opsins are unique among GPCRs because they bind an inactive form of their specific ligand, the chromophore retinal, rendering an almost instantaneous response upon light stimulation (Figure 2) (Cronin et al. 2014, Liénard et al. 2022, Varma et al. 2019). The free-retinal chromophore absorbs in the ultraviolet (UV), but its sensitivity changes when bound to an opsin to tune absorbance to specific wavelengths from the UV to the far-red portion of the visible spectrum (Cronin et al. 2014, Liénard et al. 2022). Opsins originated early in the evolution of metazoans and duplicated to give rise to three major gene groups: ciliary opsins (c-opsins) and rhabdomeric opsins (r-opsins), which are the main visual pigments of vertebrates and invertebrates, respectively, and the less-characterized retinal RGR/Go-opsins (Feuda et al. 2012, 2014). The early notion that c-opsins were restricted to vertebrates and r-opsins were exclusive to invertebrates has been revised following comparative genomic analysis (Figure 1) (Leung & Montell 2017). For example, mammalian melanopsin is related to r-opsins, and some opsins in insects are related to c-opsins (Leung & Montell 2017). In addition, other less studied opsin groups include the RGR/Go-opsins and early divergent opsins from cnidarians, ctenophores, and placozoans (Feuda et al. 2014, Leung & Montell 2017). Notably, in addition to vision, opsins are also involved in the entrainment of circadian rhythms, photorelaxation of blood vessels, and potentially temperature discrimination and audition (Leung & Montell 2017, Musilova et al. 2021). Thus, considering these unconventional roles and expression patterns, the great variation in the size of opsin repertoires across animals could underlie light-independent functions beyond visual specialization.

## 3. MECHANISMS UNDERLYING THE EVOLUTION OF SENSORY RECEPTORS

**Figure 3:**
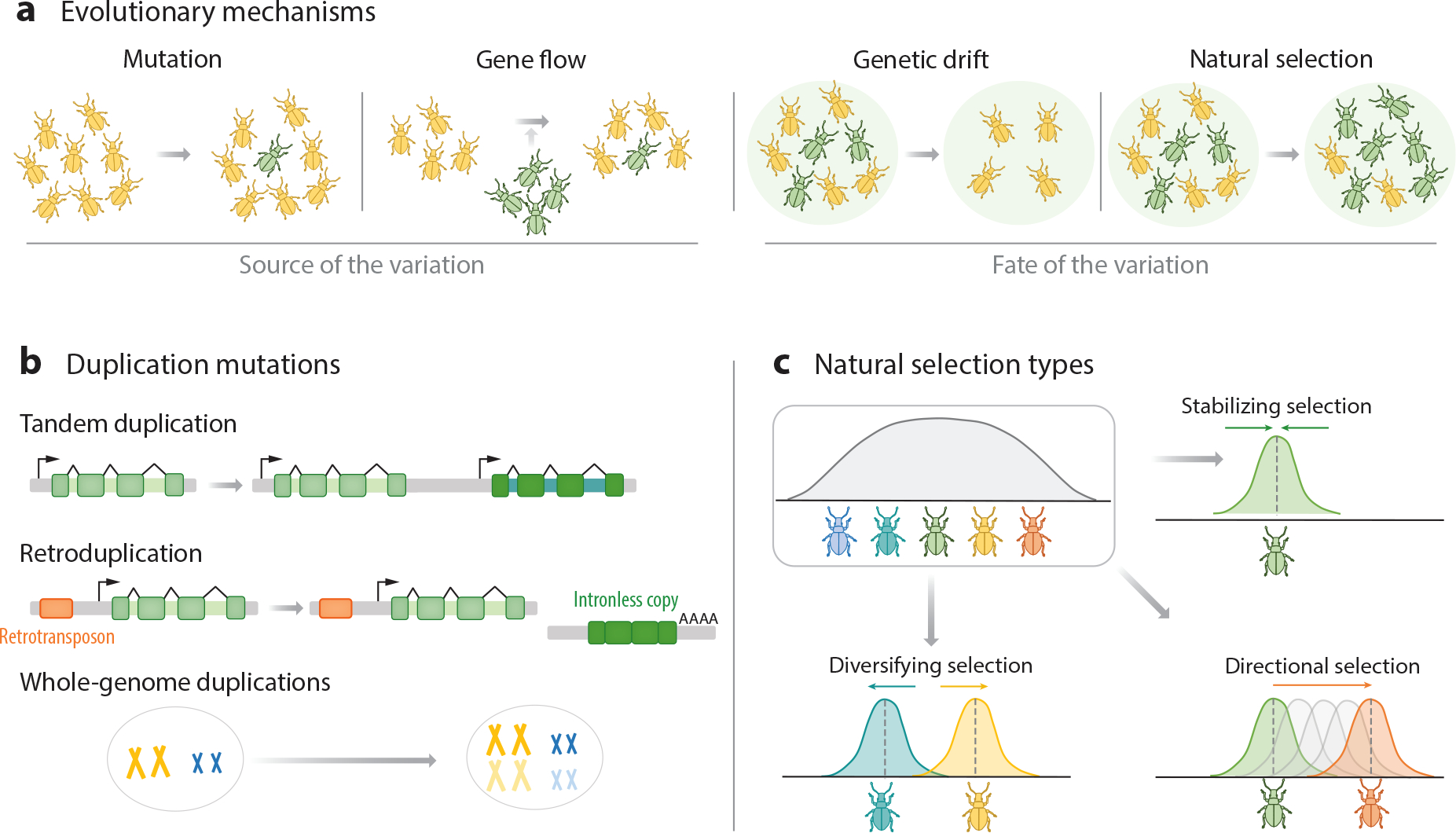
Evolutionary mechanisms at the population level drive gene diversification. (*a*) There are four main evolutionary forces. Mutation introduces new variants into the population. Gene flow introduces new variants through migration from divergent populations. Genetic drift explains the increase or decrease of variant frequency in a population due to random processes and is linked to population size. Natural selection increases the frequency of genetic variants that increase population fitness, including survival and reproduction. (*b*) Duplications are the most common type of mutation underlying the evolution of novel gene families. Tandem duplications produce identical adjacent sequences. Retroduplications result in a retrocopy of the gene devoid of introns and with a polyA tail. Whole-genome duplication entails complete chromosome duplication. (*c*) Different types of natural selection decrease, shift, or increase genetic variation in a population. Stabilizing selection decreases genetic variation, favoring an average phenotype. Directional selection favors a particular phenotype, causing the frequency of variants to continuously shift in one direction. Diversifying selection (or disruptive selection) increases genetic variation as it favors two or more phenotypes, each providing selective advantages.

How did the vast diversity of animal sensory receptors emerge? As is the case for all gene families, four main evolutionary forces orchestrate sensory receptor diversification. Mutation and gene flow introduce novel genetic variation, while genetic drift and natural selection lead to the loss or fixation of new variants (Figure 3a). Here, we describe these mechanisms of evolutionary change and highlight how they shape the adaptive function of sensory receptors.

### 3.1. Sources of Variation: Mutation and Gene Flow

Adaptive evolution is only possible if populations harbor genetic diversity. Such genetic diversity arises from genetic mutations and gene flow mediated by the migration of individuals between populations (Figure 3a). Mutations range from single-nucleotide changes and small deletions or insertions to large-scale rearrangements. Duplications are particularly critical to the evolution of novel sensory receptor families (Figure 3b) because one gene copy is rendered redundant and the duplicated gene is thus freed from selective constraints (Long et al. 2013, Nei et al. 2008). The most likely outcome of duplication is that one of the copies acquires disruptive mutations and decays into a nonfunctional pseudogene (pseudogenization) (Long et al. 2013, Roberts et al. 2022). Another outcome is that each copy may specialize in a subset of the ancestral gene functions (subfunctionalization) or that one of the copies accumulates mutations that endow it with a novel function or expression pattern (neofunctionalization) (Long et al. 2013, Roberts et al. 2022). Duplication and neofunctionalization are the primary drivers for gains of novel sensory receptor families. Interestingly, the genomic architecture of gene copies can provide insights into their duplication history. For example, tandem duplication results in a novel copy of a gene next to its progenitor (Figure 3b). They arise from unequal crossover that occurs during meiosis between misaligned homologous chromosomes, producing tandemly arranged genes (Zhang 2003). Tandem clusters of paralog genes are widespread in chemoreceptor families, including in GRs, IRs, ORs-*V*, V1Rs, and CRs, among others, highlighting the importance of this process in mediating the dynamic evolution and expression patterns of chemoreceptor genes.

Transposons, or so-called jumping genes, represent another key mechanism in the diversification of novel sensory receptor families. Retroduplication is a common type of transposition in which a gene copy transitions through an intermediate RNA in order to transpose, resulting in a new copy devoid of introns (Figure 3b). Since retrogenes insert randomly, they often lose their regulatory sequences and mostly decay into pseudogenes (Lallemand et al. 2020). However, in some cases, they can acquire novel functions. For instance, a striking number of chemoreceptors, including some groups of divergent GRs and IRs, ORs-*V*, TAARs, V1Rs, T2Rs, and CRs, are intronless, a hallmark of retroduplication. Additionally, transposable elements in the flanking regions of a gene can translocate an entire sequence, whereby the gene fragments may still contain introns. For instance, a recent study demonstrated that sex-linked UV color vision in *Heliconius* butterflies resulted from the transposition of a UV opsin from an autosome to the sex chromosome, making it obligately female-specific (Chakraborty et al. 2023).

Whole-genome duplication has also increased the sensory receptor repertoire of animals. This large-scale mutation arises from a failure of homologous chromosomes to separate during meiosis (Figure 3b) (Lallemand et al. 2020). While more common in plants, whole-genome duplications have also occurred in animals, notably twice at the base of vertebrates (Dehal & Boore 2005). Indeed, whole-genome duplication underlies the apparent single gain of an additional copy of *Piezo* in the evolutionary history of Metazoa (Figure 1). Additionally, teleosts like zebrafish have two copies of *TRPA1* that show distinct chemical and thermal sensitivity (Oda et al. 2016), and that likely emerged during a teleost whole-genome duplication event. These cases suggest that large sensory receptor genes, such as Piezo or TRP channels, might be less prone to duplication through retrotransposition or tandem duplication, with the evolution of new copies instead linked to uncommon large-scale mutations.

Finally, although mutation is the ultimate source of variation, gene flow can also introduce new variants in populations or species (Figure 3a). Gene flow between closely related species occurs through hybridization, and this transfer of genetic variation is known as introgression (Green et al. 2010, Valencia-Montoya et al. 2020). In addition, gene flow between distantly related species is also possible through diverse mechanisms of horizontal gene transfer. For instance, several lines of evidence support the eukaryote-to-prokaryote horizontal gene transfer of 7TMICs, a recently uncovered superfamily of receptors, which includes insect gustatory receptors (GRs) and odorant receptors (ORs-*I*) (Benton et al. 2020, Himmel et al. 2023). With the increasing availability of genomes and the development of new analytical frameworks to detect signatures of migration, more examples of gene flow will likely be unveiled, providing a deeper understanding of the early evolution of ancient receptor families.

### 3.2. Forces Shaping the Fate of Genetic Variation: Genetic Drift and Selection

Once a genetic variant appears through mutation or is introduced by gene flow, whether it increases or decreases in frequency depends on genetic drift and selection. Genetic drift accounts for the change in the frequency of a variant due to random chance, and its strength is tightly linked to population size (Figure 3a). A striking example of how genetic drift shapes the evolution of sensory receptor repertoires is the birth-death process of chemoreceptor families. Birth-and-death evolution occurs in multigene families when new genes arise by gene duplication, with some retained as functional genes and others inactivated by random mutations and eliminated (Nei et al. 2008). All multigene families are predicted to be subject to this mode of evolution, but chemoreceptors represent extreme cases. Even between closely related species, the number of chemoreceptor genes varies greatly, and animal genomes generally contain a large number of pseudogenes in chemosensory gene families (Figure 1) (Nei et al. 2008).

Natural selection is remarkably simple yet incredibly powerful. Natural selection can explain the exquisite structural adaptations of sensory receptors and the fit of organisms to their sensory environments. Nonetheless, it is important to recall that the emergence of variation is random and unrelated to a species’ needs. Rather, selection favors a variant that enhances survival and reproduction in a specific environment and time, and beneficial traits become more common in subsequent generations only if they are inherited. Natural selection itself encompasses three main types of selection (reviewed in Rice 2004) (Figure 3c). First, stabilizing selection, also known as purifying selection, favors an average phenotype while selecting against the extremes of a trait. Unsurprisingly, this is the most common type of selection because novel mutations that disrupt functionally relevant genes, such as sensory receptors, are generally wiped out by selection. Second, directional selection favors a particular phenotype, causing variant frequency to shift in one direction. Third, diversifying selection, or disruptive selection, increases genetic variation as it favors two or more phenotypes, each providing selective advantages. These last two types of selection that increase variation are less common but are the drivers of functional diversification and, ultimately, of the diversity of sensory systems. In the next section, we discuss methods to detect selection operating on sensory receptors.

## 4. DETECTING SELECTION IN THE EVOLUTION OF SENSORY RECEPTORS

In this section, we describe examples integrating evolutionary and sensory biology to ask interdisciplinary questions spanning molecular, cellular, and structural biology. The overarching goal is to demonstrate how applying evolutionary methods can help generate hypotheses for functional experiments that test sensory receptor adaptations. Some interdisciplinary questions include the following: Are sensory receptors under selection to drive new functions? Is there positive selection driving specific amino acid substitutions when a species shifts to a new environment? To aid researchers interested in addressing these questions, we have compiled a summary of steps and programs for comparative analysis, including those for detecting selection (Supplemental Table 1). These methods mostly use within-species diversity (population-level or microevolution) or between-species nucleotide divergence (above the species level or macroevolution) to estimate selection parameters. Within-species methods for detecting selection use population genetics approaches to identify genomic footprints left by the action of selection, such as selective sweeps. These methods allow us to detect recent signatures of selection but require sequencing the genomes of several individuals to characterize genetic variation within populations, which remains challenging for many species. Thus, the most widely used methods to infer selection exploit the ratio of synonymous substitutions (dS) and nonsynonymous (dN) substitutions. We discuss notable examples of studies that have applied these methods at different evolutionary scales and for diverse groups of sensory receptors and species.

### 4.1. Selection at the Microevolutionary Level: Selective Sweeps and Adaptive Introgression

**Figure 4:**
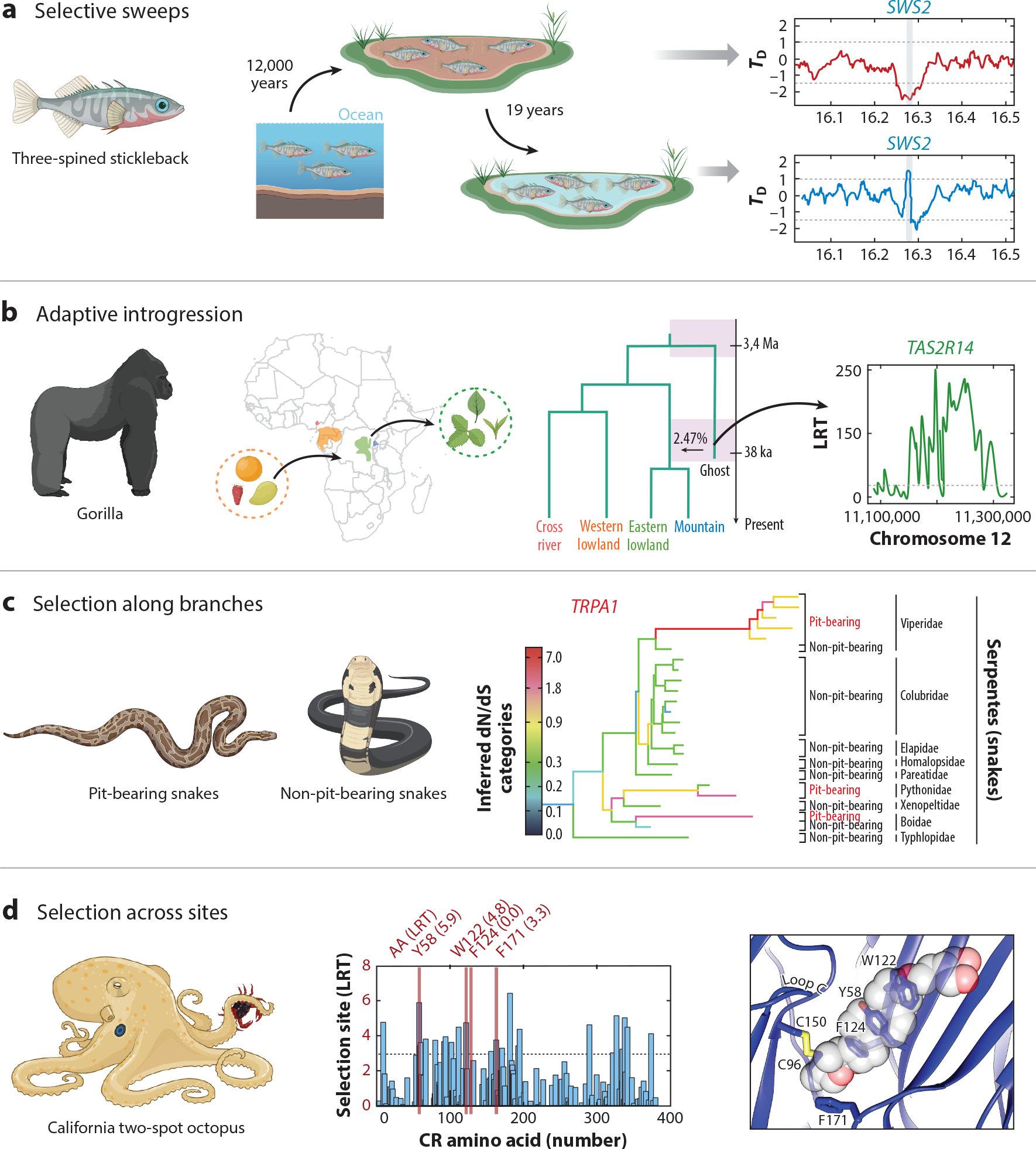
Detecting signatures of natural selection in the evolution of sensory receptors. (*a*) Selective sweep for the rapidly increased frequency of a black water–adapted blue-light-sensitive opsin (*SWS2*) variant in sticklebacks, which colonized dark water habitats after glaciation around 12,000 years ago. The black water–adapted sticklebacks were transplanted to a clear water habitat, and after 19 years, the alternate *SWS2* clear water–adapted variant increased, consistent with a transient reversed selective sweep. (*b*) Archaic admixture into eastern gorillas includes adaptive introgression (adaptive transfer of variation between species through gene flow) of a taste receptor *TAS2R14* variant. Since gorillas from eastern populations have more herbaceous diets than the frugivorous western gorillas, this introgression event likely shaped the adaptive perception of bitter taste in eastern gorillas. (*c*) TRPA1 channels underlie infrared perception in pit-bearing snakes. TRPA1 channels of pit-bearing snakes show accelerated rates of adaptive evolution compared to TRPA1 sequences of non-pit-bearing snakes. (*d*) Cephalopod CRs evolved from ancestral acetylcholine receptors. Key amino acid sites in the ligand-binding pocket of octopus CRs are under strong diversifying selection mediating the detection of hydrophobic molecules for contact-dependent aquatic chemosensation, in contrast to the ancestral detection of small polar neurotransmitters. Abbreviations: CRs, chemotactile receptors; LRT, likelihood ratio test. Panel *a* adapted from Marques et al. (2017) (CC BY 4.0). Panel *b* adapted from Pawar et al. (2023) (CC BY 4.0). Panel *c* adapted from Geng et al. (2011) (CC BY 4.0). Panel *d* adapted from Allard et al. (2023c). Illustrations of animals adapted from images created with BioRender.com.

Selective sweeps are troughs in the genetic diversity at the vicinity of a selected gene. As a positively selected variant sweeps to high frequency, this adaptive allele carries a portion of the sequence on which it arose, reducing levels of genetic diversity in the surrounding area (Booker et al. 2017). Thus, a selective sweep appears as a drop in genetic diversity because variation in this region is lost as most individuals in a population have the same advantageous variant. A stunning example of a selective sweep is found in stickleback opsins (Figure 4a). Since the retreat of the ice sheets around 12,000 years ago, marine sticklebacks have colonized hundreds of freshwater habitats (Marques et al. 2017). Some of these habitats are black water lakes, stained by dissolved tannins that lead to an almost nocturnal redshifted light environment (Marques et al. 2017). Strikingly, when analyzing genomes of three-spined sticklebacks from black water populations, Marques et al. (2017) found a selective sweep centered on the adjacent blue- and red-light-sensitive opsins *SWS2* and *LWS*. The haplotype favored in black water populations carries substitutions in the blue opsin *SWS2* that cause a redshift in light absorption. Furthermore, the authors performed a selection experiment in which sticklebacks from black water lakes were transplanted into an uninhabited clear water pond. Remarkably, after 19 years, they found that the redshifted *SWS2* opsin was disfavored in the clear water habitat of the transplant population, showing signatures of a reversed selective sweep (Marques et al. 2017). Finally, the two amino acid changes responsible for the redshift occurred independently 198 million years earlier in distantly related fish species that have also adapted to black waters (Marques et al. 2017). This study combines experimental evolution and comparative biology to reveal how adaptive changes in sensory receptors can mediate convergent evolution on different timescales.

Adaptive introgression is the transfer of adaptive genetic variation between species as a result of hybridization and repeated backcrossing. Thus, unlike a selective sweep, the adaptive variant is acquired from another population or closely related species rather than from a mutation. Until recently, the detection of adaptive introgression from genomic data relied on comparative analysis that required sequences from both recipient and donor species, such as introgression of Neanderthal and Denisovan genes into modern humans (Green et al. 2010, Reich et al. 2010). However, in many cases, the donor species is unknown (called a ghost donor population) or the genomic data are not available. Setter et al. (2020) recently developed applicable methods to detect adaptive introgression sweeps from the pattern of excess variants they produce in the flanking region of the selected gene. Pawar et al. (2023) applied this method to search for signatures of introgression in all four extant gorilla subspecies, including mountain and lowland eastern and western populations from Africa (Figure 4b). They found a signature of introgression from an archaic ghost lineage into the common ancestor of eastern gorillas but not western gorillas (Pawar et al. 2023). The adaptive introgressed regions contained the bitter taste receptor *TAS2R14*, which showed several protein-coding changes (Pawar et al. 2023). Interestingly, eastern gorillas have more herbaceous diets than frugivorous western gorillas (Pawar et al. 2023). Thus, the adaptive introgression of this receptor likely shaped the perception of bitter taste, which plays an essential role in avoiding toxic plants. Notably, this novel taste receptor variant did not result from a novel mutation or from standing variation in the western gorilla clade but was instead acquired through ancient hybridization with a now likely extinct species.

### 4.2. Selection at the Macroevolutionary Level: Selection Along Branches and Across Sites

Methods to detect purifying or diversifying selection acting on sequences primarily rely on identifying functionally relevant genes and residues, either because they are conserved or because they show lineage-specific accelerations in their evolutionary rate. The most widely used statistic for detecting selection is the dN/dS or ω ratio, which compares the rates of nonsynonymous substitutions per site (dN) with the rate of synonymous substitutions (dS) (McDonald & Kreitman 1991, Yang 2007). Because synonymous changes are neutral, their substitution rate provides a baseline against which the rate of amino acid substitution is compared (Vitti et al. 2013). Thus, a relative excess of nonsynonymous substitutions indicates diversifying or recent positive selection and will correspond to a ω ratio greater than 1 (Vitti et al. 2013). In contrast, a ω ratio smaller than 1 is indicative of purifying selection, as negative selection is acting against novel mutations (Vitti et al. 2013). The most popular tools to estimate ω ratios use codon substitution models and likelihood methods (Kosakovsky Pond et al. 2020, Yang 2007). These tools include models that can be used to detect positive selection driving adaptive protein evolution within particular lineages of a phylogeny (branch models), amino acid residues (site models), or a subset of sites along specified lineages (branch-site models) (Álvarez-Carretero et al. 2023).

Branch models are useful for detecting genes under selection as they allow for different ω ratios across a phylogeny. An example of detecting selection in sensory receptors using branch models is related to heat-seeking predation in snakes (Figure 4c). Some snakes are able to sense infrared thermal radiation using specialized pit organs that express the temperature-sensitive *TRPA1* channel (Gracheva et al. 2010). Importantly, only pit vipers, pythons, and some boas have evolved pit organs for infrared sensation (Geng et al. 2011). Remarkably, Geng et al. (2011) found that *TRPA1* is under strong positive selection in pit-bearing snakes (ω> 1) but not in other non-pit-bearing snakes and non-snake vertebrates (Geng et al. 2011). In stark contrast, *TRPV1*, a related thermoreceptor, was not under diversifying selection but instead exhibited strong purifying selection (ω < 1) with no difference between pit-bearing and non-pit-bearing snakes (Geng et al. 2011). This is an exceptional example of a tight functional link between the adaptive evolution of an ancient sensory receptor, the exquisitely sensitive organ where it is expressed, and the emergence of a specialized behavior.

In contrast to branch models, site models allow the ω ratio to vary among codons; thus, specific amino acids under selection can be identified (Álvarez-Carretero et al. 2023). A recent example where this method uncovered significant site variation is found within cephalopod CRs (Figure 4d). CRs are expressed in arm suckers, endowing octopuses with contact-dependent aquatic chemosensation of poorly soluble molecules (Allard et al. 2023c, van Giesen et al. 2020). Remarkably, site models of octopus CRs revealed numerous positively selected sites, suggesting CRs have rapidly diversified to mediate the detection of a wide array of molecules (Allard et al. 2023c). These positively selected sites are primarily concentrated in the ligand-binding pocket, and cryo-EM structures confirmed that ligand-binding residues are among those experiencing the strongest selection (Allard et al. 2023c). Therefore, octopus CRs provide one of the most comprehensive examples demonstrating the connection between adaptive substitutions in sensory receptors at the structural level and the evolution of novel organismal traits.

## 5. SENSORY RECEPTOR EVOLUTION AND DIVERSIFICATION

### 5.1. Sensory Receptors Are Hotspots for the Evolution of Reproductive Isolation

**Figure 5:**
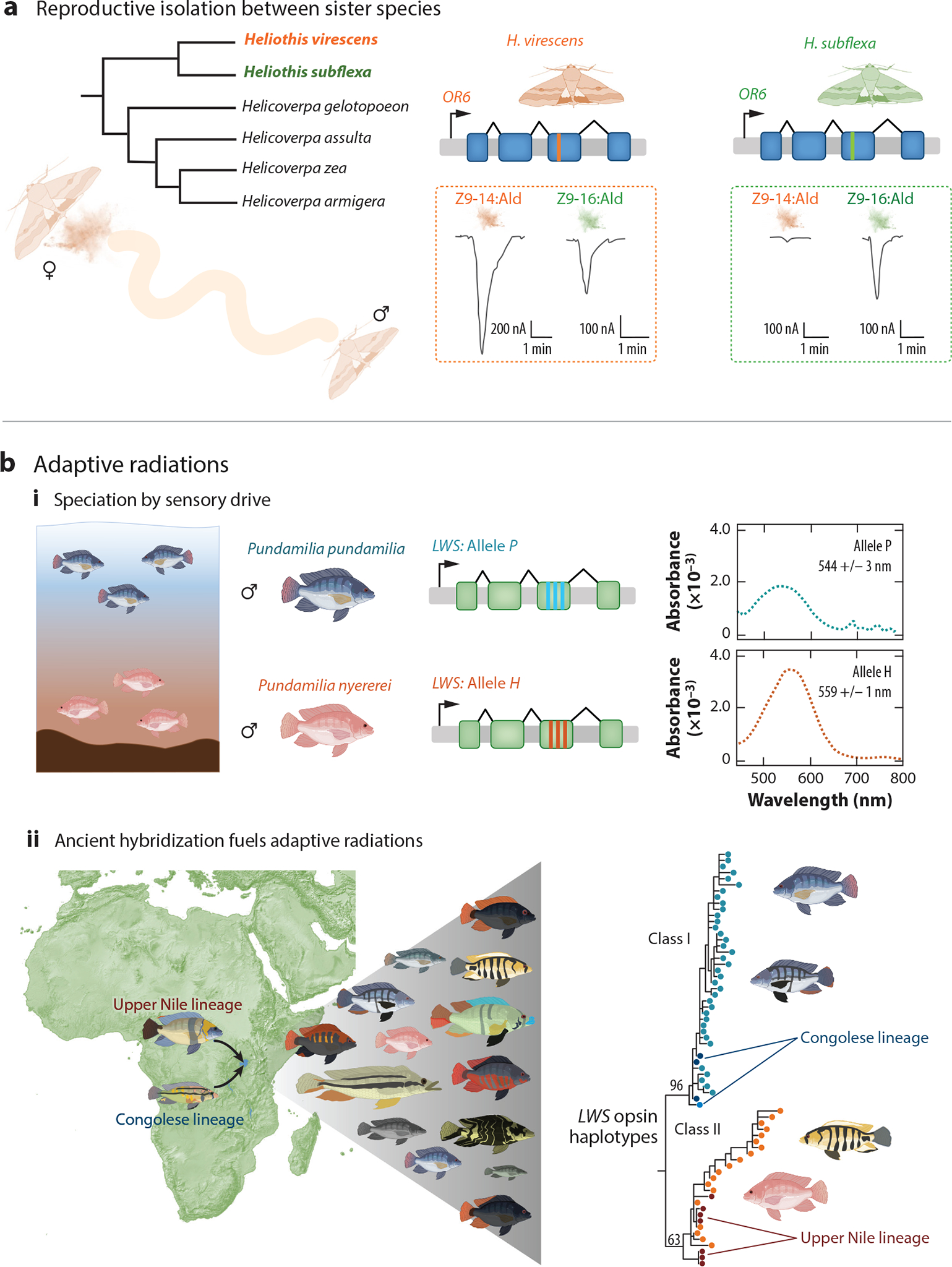
Divergence in sensory receptors drives the evolution of new species and fuels adaptive radiations. (*a*) In moths, the composition of pheromone components is highly species-specific and promotes reproductive isolation between the sister species *Heliothis virescens* and *Heliothis subflexa*. A single critical amino acid mutation in *OR6* changes receptor specificity to alter the preference of male moths for female sex pheromones of their own species. (*b*) Divergent evolution in the visual system of Lake Victoria cichlids is adapted to different depths and associated with male coloration and female preference for coloration of conspecific males, indicating reproductive isolation leading to speciation through sensory drive. (*i*) The sympatric pair of closely related cichlid species includes a red-colored species that exhibits a long-wavelength-sensitive (*LWS*) opsin haplotype adapted to the redshifted ambient light of the greater water depths it inhabits and a blue-colored species with a non-redshifted *LWS* opsin haplotype that is adapted to shallow, clear waters. (*ii*) Interspecific gene flow (hybridization) between these divergent lineages facilitated cichlid radiation by providing variation at the *LWS* locus, which is critically involved in adaptation and speciation. This case of gene flow underpinning the diversity of opsin haplotypes illustrates how evolutionary processes such as the gene flow of sensory receptor variants can facilitate adaptive radiations. Panel *a* adapted from Cao et al. (2021) (CC BY 4.0). Panel *b*, subpanel *i* adapted with permission from Seehausen et al. (2008). Panel *b*, subpanel *ii* adapted from Meier et al. (2017) (CC BY 4.0). Illustrations of animals adapted from images created with BioRender.com.

Speciation is the emergence of reproductive barriers between populations that maintain the genetic and phenotypic distinctiveness of these populations even when in geographic proximity (Seehausen et al. 2014). Since sensory receptors are required to detect signals from potential mates, they represent evolutionary hotspots for the establishment of early barriers leading to reproductive isolation (Yohe & Brand 2018). A striking example of how a single point mutation can alter sensory receptor function and driver reproductive isolation comes from studies of moth pheromone signaling (Figure 5a). Female moths produce mating pheromones that are necessary to elicit the attraction behavior of males. Indeed, male moths can track females’ pheromones from kilometers away. Notably, the composition and relative proportion of the pheromones in these secretions are highly species-specific. For example, despite being sister species, *Heliothis virescens* and *Heliothis subflexa* show divergent pheromone bouquets (Cao et al. 2021). Cao et al. (2021) cloned and functionally profiled all pheromone receptors that were associated with differences in male responses to pheromone blends and found that only orthologs of *OR6* show a different response profile between the two species. The authors found that *HvirOR6* and *HsubOR6* are narrowly tuned to the pheromone bouquets of their conspecific females. Furthermore, through site-directed mutagenesis, they show that a single point mutation (L321V) in *HvirOR6* changed its response to that of *HsubOR6* (Cao et al. 2021), tuning these odorant receptors (ORs) to their species-specific pheromone mixtures. This example underscores how even a single genetic variant in a sensory receptor can change tuning properties and affect the sensation of signals crucial for species-specific recognition and reproductive behavior.

### 5.2. Sensory Drive and Adaptive Radiations

Sensory drive predicts that selection will favor coadaptation of highly specific signals and sensory systems with respect to background noise in different environments (Stevens 2013). This is because sensory systems are shaped by the biophysical properties of distinct habitats (Stevens 2013). Consequently, receivers from a particular niche are likely to detect some stimuli better than others, resulting in biases (Cummings & Endler 2018). For example, in mate choice, males with features that match a female’s sensory bias will experience an advantage in being more easily detected, such as being smelled, seen, or heard with the greatest sensory stimulation (Cummings & Endler 2018). These biases can lead to reproductive isolation and speciation as a by-product of adaptive changes in perception and behavior based on environmental conditions (Stevens 2013).

Among the most compelling evidence for sensory drive leading to speciation is found in cichlid fish. Cichlids have undergone rapid speciation in the Great Lakes of Africa, particularly in Lake Victoria (Figure 5b). Interestingly, Lake Victoria is highly heterogeneous in ambient light because, as water depth increases, the light composition becomes more biased toward longer wavelengths (Seehausen et al. 2008). This is due to particulate matter in the water that renders the environment significantly redshifted at greater depths. Seehausen et al. (2008) tested predictions of speciation by sensory drive by studying populations of the cichlid fish *Pundamilia pundamilia* and *Pundamilia nyererei*, which are fully sympatric and live within light gradients mediated by water depth (Seehausen et al. 2008). *P. nyererei* have blue-gray male nuptial coloration (shown as the blue fish in Figure 5) and inhabit shallower waters, whereas *P. pundamilia* nuptial males are yellow and bright red colored (red fish in Figure 5) and inhabit deeper waters (Seehausen et al. 2008).

Remarkably, Seehausen et al. (2008) found that the red and blue fish predominantly possess different haplotypes (variants or alleles) of the long-wavelength-sensitive (*LWS*) opsin locus, allele *P* and allele *H*, which differ in three amino acid positions. They cloned and characterized the two haplotypes to find that allele *P* is more sensitive to shorter wavelengths and is found with high frequency in populations of the blue fish that inhabit shallow water, while allele *H* is redshifted with sensitivity to longer wavelengths and occurs more prevalently in red fish populations at greater depths (Seehausen et al. 2008). Behavioral experiments further showed that female *Pundamilia* species use male color as an important mate choice cue, exhibiting a preference for the male nuptial coloration of their own species (Seehausen et al. 2008). Thus, this example supports a critical role for sensory drive underlying speciation because the authors demonstrate functional variation in sensory receptors, an association between this variation and an environmental factor (here light among water depths), and a link between receptor allele frequency and mate choice, thereby resulting in reproductive isolation.

A relationship between opsin allele type and male coloration is also found in other cichlid species in Lake Victoria, suggesting that sensory drive may play a general role in driving cichlid speciation. Indeed, the cichlid diversity there, known as the Lake Victoria Region Superflock (LVRS), represents a classic example of adaptive radiation, as this region harbors more than 700 species that all arose rapidly in only the last 150,000 years (Figure 5b) (Meier et al. 2017). How could this huge number of species evolve on such rapid timescales? Meier et al. (2017) uncovered evidence that hybridization and gene flow between two divergent clades facilitated this process by providing genetic variation that eventually recombined and sorted into many new species (Figure 5b). Strikingly, the ancestral hybridization event generated exceptional genetic variation at the *LWS* opsin gene, which is involved in adaptation and speciation through sensory drive (Meier et al. 2017). Cichlids, therefore, serve as a particularly clear example of how evolutionary mechanisms like gene flow and hybridization generate sensory receptor diversity to facilitate rapid and extensive adaptive radiation.

## 6. SUMMARY

By analyzing the distribution of diverse sensory gene families across all major lineages of animals, we infer general patterns of sensory receptor evolution. We find that highly conserved receptors are encoded by ancient families, including TRP and Piezo channels. In contrast, chemoreceptors appear to be among the most evolutionarily labile gene families in the animal kingdom, allowing for rapid evolution among complex chemical worlds. Indeed, large-scale trade-offs resulting from losses of ancient chemoreceptor families in the two largest clades of animals likely underlie the predominance of GPCRs as sensory detectors in vertebrates, while invertebrates mostly rely on diverse groups of ion channels. In addition, we reviewed the central mechanisms of evolution and how these natural processes can account for the striking diversity of sensory systems. Collectively, these topics explore how sensory receptor evolution can provide insights into one of the core questions in biology—the origin of species.

## 7. FUTURE STUDIES OF EVOLUTIONARY SENSORY BIOLOGY

Despite recent advances in the characterization of the diversity and evolution of sensory receptors, many questions remain. To date, our knowledge of sensory receptor variation has been historically limited to model species, particularly vertebrates, and heavily biased toward identifying interspecies variation while largely neglecting variation within natural populations. Studying non-model organisms in their ecological context and moving toward characterizing within-species genetic variation will allow us to dissect the contemporary evolution of sensory receptors and elucidate how molecular adaptations influence organismal performance. Moreover, expanding analyses across phylogenetically diverse clades, including early divergent lineages and outgroups, will further uncover the ancestral sensory repertoire of animals and their role in the evolution of the nervous system. Indeed, as evidenced by the discovery of sensory receptors nested within families of neurotransmitters, the number of peripheral sensory receptors that evolved from other neuron-specific receptors is likely to be greater than currently recognized. Similarly, novel thermosensory receptors could be nested within families of chemoreceptors, as these modalities seem tightly linked over evolutionary time. Lastly, investigating convergence of sensory functions at the scale of the animal tree will provide a robust comparative framework to probe for constraints in sensory integration and the predictability of evolution.

Novel approaches will continue to shape our understanding of sensory receptors. The cryoEM structural revolution, further fostered by inference using AlphaFold, will continue to provide increased power to contrast predictions of selected sites in receptor proteins and functionally relevant adaptations. Furthermore, improved bioinformatic methods that consider three-dimensional structural motifs can help delineate the deep evolutionary history of receptor families, including those in the so-called twilight zone of sequence similarity (Benton et al. 2020, Himmel et al. 2023). Nevertheless, protein biochemistry and physiology will remain foundational and necessary approaches for connecting evolutionary analyses, structural biology, and organismal function. Finally, a promising avenue for future research is to understand how complex evolutionary interactions between distinct receptor types, sensory modalities, and processing mechanisms synergistically give rise to diverse animal behavior.

## Supplementary Material

Supplementary Evolution of Sensory Receptors
