# Supplementary material for: Evolution of Sensory Receptors
Source: Annu Rev Cell Dev Biol. Author manuscript; Available in PMC 2024 Oct 31. (PMC11526382; doi:10.1146/annurev-cellbio-120123-112853)
Supplement: Supplementary Evolution of Sensory Receptors [file NIHMS2029053-supplement-Supplementary_Evolution_of_Sensory_Receptors.docx]

**Evolution of sensory receptors**

**Supplemental Appendix**

Wendy A. Valencia-Montoya^1,2^, Naomi E. Pierce^1^, Nicholas W. Bellono^2^

*^1^ Department of Organismic and Evolutionary Biology and Museum of Comparative Zoology, Harvard University, Cambridge, MA, USA*

*^2^ Department of Molecular and Cell Biology, Harvard University, Cambridge, MA, USA*

^#^*Correspondence:* [*wvalenciamontoya@g.harvard.edu*](mailto:wvalenciamontoya@g.harvard.edu) *and* [*nbellono@harvard.edu*](mailto:nbellono@harvard.edu)

**Supplemental Table 1.** Programs for evolutionary analysis, highlighting tools to detect natural selection.

| Use | Suggested programs or pipelines |
| --- | --- |
| Characterizing variation in sensory receptors | **Multiple sequence alignments:** MAFFT (Katoh & Standley 2013), MUSCLE (Edgar 2004).  **Mapping to reference sequences:** BWA (Li & Durbin 2009), Bowtie (Langmead et al. 2009), STAR (Dobin et al. 2013).  **Variant calling:** GATK (DePristo et al. 2011), bcftools (Li 2011), FreeBayes (Garrison & Marth 2012) |
| Detecting natural selection | ***Within-species:***  **Outlier analyses (OA):** ARLEQUIN (Excoffier et al. 2005), FDIST (Beaumont & Nichols 1996), BAYESCAN (Foll & Gaggiotti 2008).  **Selective sweeps:** SweepFinder2 (DeGiorgio et al. 2016) . SweedD (Pavlidis et al. 2013). Flex-sweep (Lauterbur et al. 2023).  **Adaptive introgression*:*** *f*-statistics (Green et al. 2010), VolcanoFinder (Setter et al. 2020). |
|  | ***Between-species and for diverged sequences:***  **dS/dN ratio:** PAML (Yang 2007). HyPhy (Kosakovsky Pond et al. 2020) |
| Structural analyses | Alpha-Fold (Jumper et al. 2021), SWISS-MODEL (Waterhouse et al. 2018), PyMOL (PyMOL 2021), UCFS Chimera-A (Pettersen et al. 2004). |

**Sensory receptor survey**

We used recent phylogenetic hypotheses of major animal clades to map gains and losses of sensory receptor families (Dunn et al. 2014; Himmel et al. 2020; Schultz et al. 2023). Our concept of the early evolution of Metazoa, which contains most animals as we understand them today, has been shifting due to the discovery of new taxa as well as different methodological approaches for tree inference (Dunn et al. 2014; Himmel et al. 2020; Schultz et al. 2023). Here, animals are considered as synonymous of Metazoa, following (Dunn et al. 2014; Himmel et al. 2020; Schultz et al. 2023). We infer gains and losses primarily based in the literature, as well as on a general survey we carried for exemplar taxa.

To retrieve coding sequences of sensory receptors, we leveraged whole genomes with protein annotations on the Genbank for species placeholders from all major animal clades (**Table S2**). As chemoreceptors have been widely studied, repertoire sizes were mostly retrieved from literature (Baldwin & Ko 2020; Churcher & Taylor 2011; Eyun et al. 2017; Guo et al. 2022; Himmel & Cox 2020; Kadowaki 2015; Leung & Montell 2017; Nei et al. 2008; Ni 2021; Policarpo et al. 2023; Robertson 2019; Saito & Tominaga 2015; Sparks et al. 2018; Vidal et al. 2018; Wicher & Miazzi 2021). When a literature record was not found for a particular group, we used iterative search in jackHMMER (Eddy 1998; Finn et al. 2011) as described below and confirmed sequence identity by blasting against the NCBI nr database for the species of interest (Madden 2003). We focused on well-studied groups, although there are putative receptor families that await validation and study. For example, *Nematostella vectensis* exhibits an expansion of OR-like genes with similar structure to vertebrates ORs (Churcher & Taylor 2011). In Figure 1, we included these genes as OR-*V*s following Baldwin & Ko 2020 and Churcher & Taylor 2011. Because of the sparse pattern of presence/absence of OR-*V*s across non-vertebrate groups, we note that they might represent an independent expansion. Thus, a more focused phylogenetic survey of cnidarians OR-like receptors could shed further light on the antiquity and specificity of this group and its relation to canonical OR-*V* receptors. There are likely additional similar examples.

We then focused on mining mechanoreceptors, thermoreceptors, and light receptors. We used known sensory receptors as well as outgroup sequences as protein templates (**Table S3-6**). Sensory receptors were mined using these queries for iterative search in jackHMMER (Eddy 1998; Finn et al. 2011) with a stringent e-value of 10e-30, following (Himmel et al. 2020; Peng et al. 2015). To curate and improve the quality of the initial database, protein sequences were clustered at 90% identity using CD-hit (Li & Godzik 2006) to remove isoforms and duplicates, retaining the longest isoform in order to maximize available phylogenetic information. We then predicted Transmembrane Topology (TM) and excluded sequences which did not have the predicted number of TM segments characteristic of each gene family using Phobius (Käll et al. 2007). We generated amino acid sequence alignments for each gene family, including queries and outgroups, with MAFFT v.74 (Katoh & Standley 2013). Finally, we used ModelFinder (Kalyaanamoorthy et al. 2017) to assess the best model of substitution for phylogenetic inference.

Queries and retrieved sequences were used to estimate maximum likelihood gene trees in IQ-TREE v2.0 (Minh et al. 2020) to confirm sensory receptor family and subfamily identity. Phylogenies were visualized in Figtree v.1.4.4. (*FigTree*) and “ggtree” v.3.4.252 (Yu et al. 2017). Sequences that clustered with known sensory receptor genes in the phylogenetic tree and had a length equal or higher than the mean expected gene length for the group, were kept. In addition to the phylogenetic evidence, we confirmed that the predicted sequences were best-matching against known sensory receptor genes for the distinct families by using the retrieved sequences as queries in blastp (Madden 2003) search against the NCBI nr database. Gains and losses of gene families throughout the animal phylogeny were mapped primarily based on literature and the most early divergent group whereby a complete coding sequence was recovered.

**Supplemental Table 2.** Table of reference genomes of species placeholders included in receptor analysis.

| **Group** | **Scientifc name** | **Genus** | **Species** | **RefSeq** |
| --- | --- | --- | --- | --- |
| Filasteria | *Capsaspora owczarzaki* | *Capsaspora* | *owczarzaki* | GCF_000151315.2 |
| Choanoflagellates | *Salpingoeca rosetta* | *Salpingoeca* | *rosetta* | GCF_000188695.1 |
| Porifera | *Amphimedon queenslandica* | *Amphimedon* | *queenslandica* | GCF_000090795.2 |
| Placozoa | *Trichoplax adhaerens* | *Trichoplax* | *adhaerens* | GCF_000150275.1 |
| Hydrozoa | *Hydra vulgaris* | *Hydra* | *vulgaris* | GCF_022113875.1 |
| Anthozoa | *Nematostella vectensis* | *Nematostella* | *vectensis* | GCF_932526225.1 |
| Echinodermata | *Strongylocentrotus purpuratus* | *Strongylocentrotus* | *purpuratus* | GCF_000002235.5 |
| Hemichordata | *Saccoglossus kowalevskii* | *Saccoglossus* | *kowalevskii* | GCF_000003605.2 |
| Tunicata | *Ciona intestinalis* | *Ciona* | *intestinalis* | GCF_000224145.3 |
| Cephalochordata | *Branchiostoma floridae* | *Branchiostoma* | *floridae* | GCF_000003815.2 |
| Agnatha | *Petromyzon marinus* | *Petromyzon* | *marinus* | GCF_010993605.1 |
| Chondrichthyes | *Carcharodon carcharias* | *Carcharodon* | *carcharias* | GCF_017639515.1 |
| Actinopterygii | *Danio rerio* | *Danio* | *rerio* | GCF_000002035.6 |
| Coelacanthiformes | *Latimeria chalumnae* | *Latimeria* | *chalumnae* | GCF_000225785.1 |
| Dipnomorpha | *Protopterus annectens* | *Protopterus* | *annectens* | GCF_019279795.1 |
| Mammalia | *Mus musculus* | *Mus* | *musculus* | GCF_000001635.27 |
| Mammalia | *Homo sapiens* | *Homo* | *sapiens* | GCF_000001405.40 |
| Amphibia | *Xenopus laevis* | *Xenopus* | *laevis* | GCF_017654675.1 |
| Aves | *Gallus gallus* | *Gallus* | *gallus* | GCF_016699485.2 |
| Testudines | *Chelonia mydas* | *Chelonia* | *mydas* | GCF_015237465.2 |
| Crocodylia | *Alligator mississippiensis* | *Alligator* | *mississippiensis* | GCF_000281125.3 |
| Lepidosauria | *Anolis carolinensis* | *Anolis* | *carolinensis* | GCF_000090745.1 |
| Cephalopoda | *Octopus bimaculoides* | *Octopus* | *bimaculoides* | GCF_001194135.2 |
| Gastropoda | *Aplysia californica* | *Aplysia* | *californica* | GCF_000002075.1 |
| Nemertea | *Lingula anatina* | *Lingula* | *anatina* | GCF_001039355.2 |
| Phoronida | *Priapulus caudatus* | *Priapulus* | *caudatus* | GCF_000485595.1 |
| Nematoda | *Caenorhabditis elegans* | *Caenorhabditis* | *elegans* | GCF_000002985.6 |
| Tardigrada | *Paramacrobiotus metropolitanus* | *Paramacrobiotus* | *metropolitanus* | GCF_019649055.1 |
| Xiphosura | *Limulus polyphemus* | *Limulus* | *polyphemus* | GCF_000517525.1 |
| Arachnida | *Ixodes scapularis* | *Ixodes* | *scapularis* | GCF_016920785.2 |
| Crustacea | *Daphnia pulex* | *Daphnia* | *pulex* | GCF_021134715.1 |
| Insecta | *Drosophila melanogaster* | *Drosophila* | *melanogaster* | GCF_000001215.4 |
| Insecta | *Tribolium castaneum* | *Tribolium* | *castaneum* | GCF_000002335.3 |
| Insecta | *Acyrthosiphon pisum* | *Acyrthosiphon* | *pisum* | GCF_005508785.2 |

**Supplemental Table 3.** Sequence queries, including GenBank accession numbers, used to mine Piezo proteins.

| **Clade** | **Species** | **GenBank Accession** |
| --- | --- | --- |
| Piezo | *Nematostella vectensis* | XP_048576630.1 |
| Piezo | *Drosophila melanogaster* | AFB77909.1 |
| Piezo1 | *Danio rerio* | XP_696355.4 |
| Piezo2 | *Danio rerio* | XP_021323945.1 |
| Piezo1 | *Mus musculus* | ADN28064.1 |
| Piezo2 | *Mus musculus* | ADN28065.1 |

**Supplemental Table 4.** Sequence queries, including GenBank accession numbers, used to mine and annotate Transient Receptor Potential (TRP) ion channels. For TRP channels group: TRPP (polycystin or polycystic kidney disease), TRPML (mucolipin), TRPA (ankyrin), TRPV (vanilloid), TRPVL (vanilloid-like), TRPC (canonical), TRPN (nompC, or no mechanoreceptor potential C), TRPM (melastatin). Outgroups correspond to Shaker potassium channels. These sequences were selected from (Peng et al. 2015).

| **Clade** | **Name in Peng et al. 2015** | **GenBank Accession** |
| --- | --- | --- |
| TRPA | mTRPA1 | NP_808449.1 |
| TRPC | mTRPC1 | NP_035773.1 |
| TRPC | mTRPC2 | NP_001103367.1 |
| TRPC | mTRPC3 | NP_062383.2 |
| TRPC | mTRPC4 | NP_001240611.1 |
| TRPC | mTRPC5 | NP_033454.1 |
| TRPC | mTRPC6 | NP_038866.2 |
| TRPC | mTRPC7 | NP_036165.1 |
| TRPM | mTRPM1 | NP_001034193.2 |
| TRPM | mTRPM2 | NP_612174.2 |
| TRPM | mTRPM3 | NP_001030320.1 |
| TRPM | mTRPM4 | NP_780339.2 |
| TRPM | mTRPM5 | NP_064673.2 |
| TRPM | mTRPM6 | NP_700466.1 |
| TRPM | mTRPM7 | NP_001157797.1 |
| TRPM | mTRPM8 | NP_599013.1 |
| TRPML | mTRPML1 | NP_444407.1 |
| TRPML | mTRPML2 | NP_080932.2 |
| TRPML | mTRPML3 | NP_598921.1 |
| TRPP | mTRPP2 | NP_032887.3 |
| TRPP | mTRPP3 | NP_852087.2 |
| TRPP | mTRPP5 | NP_058623.2 |
| TRPV | mTRPV1 | NP_001001445.1 |
| TRPV | mTRPV2 | NP_035836.2 |
| TRPV | mTRPV3 | NP_659567.2 |
| TRPV | mTRPV4 | NP_071300.2 |
| TRPV | mTRPV5 | NP_001007573.1 |
| TRPV | mTRPV6 | NP_071858.2 |
| TRPN | CeTRP-4 | CAC14420.3 |
| TRPN | DrTRPN1 | NP_899192.1 |
| TRPA | DmTRPA1 | NP_648263.5 |
| TRPA | DmPain | NP_611979.1 |
| TRPA | DmPyx | NP_612015.1 |
| TRPA | DmWtrw | NP_731193.1 |
| TRPM | DmTRPM | NP_001036548.1 |
| TRPML | DmTRPML | NP_649145.1 |
| TRPN | DmNompC | NP_523483.1 |
| TRPP | DmPkd2 | NP_609561.2 |
| TRPV | DmIav | NP_572353.1 |
| TRPV | DmNan | NP_001261833.1 |
| TRPA | NvPain | XP_001601841.2 |
| TRPA | NvPyx | XP_001600001.1 |
| TRPA | NvTRPA5 | NW_001818681.1 |
| TRPA | NvHsTRPA | XP_001604057.1 |
| TRPA | Nvwtrw | XP_003427362.1 |
| TRPM | NvTRPM | XP_001600197.1 |
| TRPML | NvTRPML | XP_001606145.2 |
| TRPN | NvNompC | XP_001605939.2 |
| TRPV | NvIav | XP_001602588.2 |
| TRPV | NvNan | XP_001606102.2 |
| Outgroup | DmShaker | CAA29917.1 |
| Outgroup | TcShaker | XP_001809693.1 |
| Outgroup | AmShaker | XP_391895.3 |
| Outgroup | PhShaker | XP_002422887.1 |
| Outgroup | ApShaker | XP_001947567.2 |
| Outgroup | mShaker | NP_067250.2 |

**Supplemental Table 5.** Sequence queries, including GenBank accession numbers, used to mine and annotate opsins, including C-opsins (ciliary opsins), R-opsins (rhabdomeric opsins), RGR-Go opsins (retinal G-protein couple receptor), cnidarians (cnidopsins), and placozoan (placopsins) specific opsins. Outgroups correspond to Melatonin receptors. These sequences were selected from (Feuda et al. 2012).

| **Opsin group** | **Name in Feuda et al. 2012** | **GenBank Accession** |
| --- | --- | --- |
| RGR-Go opsins | 026NeuBota_ | NP_001193009 |
| RGR-Go opsins | 024NeuMumu_ | EDL23393 |
| RGR-Go opsins | 027NeuPatr_ | XP_001146167.1 |
| RGR-Go opsins | 032NeuModo_ | XP_001369202 |
| RGR-Go opsins | 021NeuDare_ | NP_001186975.1 |
| RGR-Go opsins | 018PerMumu | NP_033128.1 |
| RGR-Go opsins | 019PerDare | NP_001004654.1 |
| RGR-Go opsins | 010RGRDare_ | NP_001017877.1 |
| Cnidopsins | CL_CN168_A | BAF95836.1 |
| Cnidopsins | ClR_CN120S | BAF95833.1 |
| Cnidopsins | HM_CN170_U | XP_002160448.1 |
| Cnidopsins | CR_CN100_K | BAG80696.1 |
| Cnidopsins | Plos1_2_1- | FAA00390 |
| Cnidopsins | NV_CN158Su | FAA00389.1 |
| C-opsins | 173RHOMumu_ | NP_663358.1 |
| C-opsins | 156RHOLeja_ | P22671.1 |
| C-opsins | 148RHOGaga_ | NP_990821.1 |
| C-opsins | 211SWSDare_ | NP_571267.1 |
| C-opsins | 128SWSPore_ | ABB69699.1 |
| C-opsins | 043uppTeni_ | CAG06878.1 |
| C-opsins | 041ParXetr_ | NP_001039256.1 |
| C-opsins | OG45ApimeT | NP_001035057.1 |
| R-opsins | 204NeooeL1_ | ACU00210.1 |
| R-opsins | 045AnogaRE_ | XP_001238571.2 |
| R-opsins | 004HelerRE_ | AAY16540 |
| R-opsins | 077DromeOC_ | NP_524398.1 |
| R-opsins | 165BrakuM1_ | BAG80990.1 |
| R-opsins | 120PieraUV_ | BAE19944.1 |
| R-opsins | 150DappuU1_ | EFX81332.1 |
| R-opsins | OG7PlatynP_ | CAC86665.1 |
| Cnidopsins | NV_CN151Su | FAA00396.1 |
| Cnidopsins | NV_CN146Su | FAA00401.1 |
| Placopsins | Tra_429091jgi\|T | XP_002112437.1 |
| Placopsins | Tra_430364jgi\|T | XP_002113363.1 |
| Placopsins | Tra_429091jgi\|T | XP_002112437.1 |
| Outgroup | Tra_435656jgi\|T | XP_002114590.1 |
| Outgroup | Tra_434217jgi\|T | XP_002109502.1 |
| Outgroup | Tra_435668jgi\|T | XP_002114593.1 |
| Outgroup | TRA_530235_jgi\| | XP_002114830.1 |
| Outgroup | NEM_445570_jgi\| | XP_001631194.1 |

**Supplemental Table 6.** Sequence queries used to mine and annotate homologs of Gustatory receptors (GRs). These sequences were selected from (Saina et al. 2015). Note that here we are referring to Gustatory-like receptors (Grl) following Saina et al. 2015, but in the in the review in Fig. 1, gustatory-like receptors are referred globally as the Metazoan GRs.

| **Group** | **Name in Saina et al. 2015** | **Accession ID** |
| --- | --- | --- |
| Grl | CtelGrl1 | ELT92320 |
| Grl | CtelGrl2 | ELT92788 |
| Grl | CtelGrl3 | ELT90076 |
| Grl | CtelGrl4 | ELU10797 |
| Grl | CtelGrl5 | ELT90075 |
| Grl | CtelGrl6 | ELU14052 |
| Grl | CtelGrl7 | ELT97787 |
| Grl | CtelGrl8 | ELU10343 |
| Grl | CtelGrl9 | ELT92997 |
| Grl | CtelGrl10 | ELT90557 |
| Grl | CtelGrl11 | ELU06252 |
| Grl | CtelGrl12 | ELU13825 |
| Grl | HrobGrl1 | 163318 |
| Grl | HrobGrl2 | 163317 |
| Grl | LgigGrl1 | 168741 |
| Grl | LgigGrl2 | 171766 |
| Grl | LgigGrl3 | 152656 |
| Grl | LgigGrl4 | 159388 |
| Grl | LgigGrl5 | 172086 |
| Grl | LgigGrl6 | 160675 |
| Grl | LgigGrl7 | 159430 |
| Grl | CgigGrl1 | EKC21365 |
| Grl | CgigGrl2 | EKC32937 |
| Grl | AcalGrl1 | XP_005105989.1 |
| Grl | AcalGrl2 | XP_005105990.1 |
| Grl | AcalGrl3 | XP_005099561.1 |
| Grl | SkowGrl1 | XM_002731392 |
| Grl | SpurGrl1 | XM_003730711 |
| Grl | SpurGrl2 | Ctg127547 |
| Grl | SpurGrl3 | Ctg127545-2 |
| Grl | SpurGrl4 | Ctg127545-1 |
| Grl | SpurGrl5 | Ctg131000 |
| Grl | LvarGrl1 | Contig203256 |
| Grl | LvarGrl2 | Contig339833 |
| Grl | LvarGrl3 | Contig203252 |
| Grl | LvarGrl4 | Contig122808 |
| Grl | LvarGrl5 | Contig122810 |
| Grl | LvarGrl6 | Contig203253 |
| Grl | LvarGrl7 | Contig17632 |
| Grl | PminGrl1 | HP136897.1 |
| Grl | AmilGrl1 | EZ016165.1 |
| Grl | NvecGrl1 | KP294348 |
| Grl | NvecGrl2 | KP294349 |
| Grl | TadhGrl1 | XM_002117218 |
| Grl | TadhGrl2 | XM_002110287 |
| Grl | TadhGrl3 | XM_002110288 |

**References**

Baldwin MW, Ko M-C. 2020. Functional evolution of vertebrate sensory receptors. *Hormones and Behavior*. 124:104771

Beaumont MA, Nichols RA. 1996. Evaluating loci for use in the genetic analysis of population structure. *Proceedings of the Royal Society of London. Series B: Biological Sciences*. 263(1377):1619–26

Churcher AM, Taylor JS. 2011. The Antiquity of Chordate Odorant Receptors Is Revealed by the Discovery of Orthologs in the Cnidarian Nematostella vectensis. *Genome Biology and Evolution*. 3:36–43

DeGiorgio M, Huber CD, Hubisz MJ, Hellmann I, Nielsen R. 2016. SweepFinder2: increased sensitivity, robustness and flexibility. *Bioinformatics*. 32(12):1895–97

DePristo MA, Banks E, Poplin R, Garimella KV, Maguire JR, et al. 2011. A framework for variation discovery and genotyping using next-generation DNA sequencing data. *Nat Genet*. 43(5):491–98

Dobin A, Davis CA, Schlesinger F, Drenkow J, Zaleski C, et al. 2013. STAR: ultrafast universal RNA-seq aligner. *Bioinformatics*. 29(1):15–21

Dunn CW, Giribet G, Edgecombe GD, Hejnol A. 2014. Animal Phylogeny and Its Evolutionary Implications. *Annu. Rev. Ecol. Evol. Syst.* 45(1):371–95

Eddy SR. 1998. Profile hidden Markov models. *Bioinformatics*. 14(9):755–63

Edgar RC. 2004. MUSCLE: multiple sequence alignment with high accuracy and high throughput. *Nucleic Acids Res*. 32(5):1792–97

Excoffier L, Laval G, Schneider S. 2005. Arlequin (version 3.0): An integrated software package for population genetics data analysis. *Evol Bioinform Online*. 1:117693430500100003

Eyun S, Soh HY, Posavi M, Munro JB, Hughes DST, et al. 2017. Evolutionary History of Chemosensory-Related Gene Families across the Arthropoda. *Molecular Biology and Evolution*. 34(8):1838–62

Feuda R, Hamilton SC, McInerney JO, Pisani D. 2012. Metazoan opsin evolution reveals a simple route to animal vision. *PNAS*. 109(46):18868–72

*FigTree*. http://tree.bio.ed.ac.uk

Finn RD, Clements J, Eddy SR. 2011. HMMER web server: interactive sequence similarity searching. *Nucleic Acids Res*. 39(Web Server issue):W29–37

Foll M, Gaggiotti O. 2008. A genome-scan method to identify selected loci appropriate for both dominant and codominant markers: a Bayesian perspective. *Genetics*. 180(2):977–93

Garrison E, Marth G. 2012. Haplotype-based variant detection from short-read sequencing. *arXiv:1207.3907 [q-bio]*

Green RE, Krause J, Briggs AW, Maricic T, Stenzel U, et al. 2010. A Draft Sequence of the Neandertal Genome. *Science*. 328(5979):710–22

Guo L, Dai W, Xu Z, Liang Q, Miller ET, et al. 2022. Evolution of Brain-Expressed Biogenic Amine Receptors into Olfactory Trace Amine-Associated Receptors. *Molecular Biology and Evolution*. 39(3):msac006

Himmel NJ, Cox DN. 2020. Transient receptor potential channels: current perspectives on evolution, structure, function and nomenclature. *Proc. R. Soc. B.* 287(1933):20201309

Himmel NJ, Gray TR, Cox DN. 2020. Phylogenetics Identifies Two Eumetazoan TRPM Clades and an Eighth TRP Family, TRP Soromelastatin (TRPS). *Molecular Biology and Evolution*. 37(7):2034–44

Jumper J, Evans R, Pritzel A, Green T, Figurnov M, et al. 2021. Highly accurate protein structure prediction with AlphaFold. *Nature*. 596(7873):583–89

Kadowaki T. 2015. Evolutionary dynamics of metazoan TRP channels. *Pflugers Arch - Eur J Physiol*. 467(10):2043–53

Käll L, Krogh A, Sonnhammer ELL. 2007. Advantages of combined transmembrane topology and signal peptide prediction--the Phobius web server. *Nucleic Acids Res*. 35(Web Server issue):W429-432

Kalyaanamoorthy S, Minh BQ, Wong TKF, von Haeseler A, Jermiin LS. 2017. ModelFinder: fast model selection for accurate phylogenetic estimates. *Nature Methods*. 14(6):587–89

Katoh K, Standley DM. 2013. MAFFT Multiple sequence alignment software version 7: improvements in performance and usability. *Mol Biol Evol*. 30(4):772–80

Kosakovsky Pond SL, Poon AFY, Velazquez R, Weaver S, Hepler NL, et al. 2020. HyPhy 2.5—A Customizable Platform for Evolutionary Hypothesis Testing Using Phylogenies. *Molecular Biology and Evolution*. 37(1):295–99

Langmead B, Trapnell C, Pop M, Salzberg SL. 2009. Ultrafast and memory-efficient alignment of short DNA sequences to the human genome. *Genome Biology*. 10(3):R25

Lauterbur ME, Munch K, Enard D. 2023. Versatile Detection of Diverse Selective Sweeps with Flex-Sweep. *Molecular Biology and Evolution*. 40(6):msad139

Leung NY, Montell C. 2017. Unconventional Roles of Opsins. *Annu. Rev. Cell Dev. Biol.* 33(1):241–64

Li H. 2011. A statistical framework for SNP calling, mutation discovery, association mapping and population genetical parameter estimation from sequencing data. *Bioinformatics*. 27(21):2987–93

Li H, Durbin R. 2009. Fast and accurate short read alignment with Burrows–Wheeler transform. *Bioinformatics*. 25(14):1754–60

Li W, Godzik A. 2006. Cd-hit: a fast program for clustering and comparing large sets of protein or nucleotide sequences. *Bioinformatics*. 22(13):1658–59

Madden T. 2003. *The BLAST Sequence Analysis Tool*. National Center for Biotechnology Information (US)

Minh BQ, Schmidt HA, Chernomor O, Schrempf D, Woodhams MD, et al. 2020. IQ-TREE 2: New Models and Efficient Methods for Phylogenetic Inference in the Genomic Era. *Molecular Biology and Evolution*. 37(5):1530–34

Nei M, Niimura Y, Nozawa M. 2008. The evolution of animal chemosensory receptor gene repertoires: roles of chance and necessity. *Nat Rev Genet*. 9(12):951–63

Ni L. 2021. The Structure and Function of Ionotropic Receptors in Drosophila. *Front. Mol. Neurosci.* 13:638839

Pavlidis P, Živković D, Stamatakis A, Alachiotis N. 2013. SweeD: Likelihood-Based Detection of Selective Sweeps in Thousands of Genomes. *Molecular Biology and Evolution*. 30(9):2224–34

Peng G, Shi X, Kadowaki T. 2015. Evolution of TRP channels inferred by their classification in diverse animal species. *Molecular Phylogenetics and Evolution*. 84:145–57

Pettersen EF, Goddard TD, Huang CC, Couch GS, Greenblatt DM, et al. 2004. UCSF Chimera—A visualization system for exploratory research and analysis. *Journal of Computational Chemistry*. 25(13):1605–12

Policarpo M, Baldwin M, Casane D, Salzburger W. 2023. Diversity and evolution of the vertebrate chemoreceptor gene repertoire. In Review

PyMOL. 2021. The PyMOL Molecular Graphics System, Version 1.2r3pre, Schrödinger, LLC.

Robertson HM. 2019. Molecular Evolution of the Major Arthropod Chemoreceptor Gene Families. *Annu. Rev. Entomol.* 64(1):227–42

Saina M, Busengdal H, Sinigaglia C, Petrone L, Oliveri P, et al. 2015. A cnidarian homologue of an insect gustatory receptor functions in developmental body patterning. *Nat Commun*. 6(1):6243

Saito S, Tominaga M. 2015. Functional diversity and evolutionary dynamics of thermoTRP channels. *Cell Calcium*. 57(3):214–21

Schultz DT, Haddock SHD, Bredeson JV, Green RE, Simakov O, Rokhsar DS. 2023. Ancient gene linkages support ctenophores as sister to other animals. *Nature*. 618(7963):110–17

Setter D, Mousset S, Cheng X, Nielsen R, DeGiorgio M, Hermisson J. 2020. VolcanoFinder: Genomic scans for adaptive introgression. *PLoS Genet*. 16(6):e1008867

Sparks JT, Botsko G, Swale DR, Boland LM, Patel SS, Dickens JC. 2018. Membrane Proteins Mediating Reception and Transduction in Chemosensory Neurons in Mosquitoes. *Front. Physiol.* 9:1309

Vidal B, Aghayeva U, Sun H, Wang C, Glenwinkel L, et al. 2018. An atlas of Caenorhabditis elegans chemoreceptor expression. *PLOS Biology*. 16(1):e2004218

Waterhouse A, Bertoni M, Bienert S, Studer G, Tauriello G, et al. 2018. SWISS-MODEL: homology modelling of protein structures and complexes. *Nucleic Acids Res*. 46(W1):W296–303

Wicher D, Miazzi F. 2021. Functional properties of insect olfactory receptors: ionotropic receptors and odorant receptors. *Cell Tissue Res*. 383(1):7–19

Yang Z. 2007. PAML 4: phylogenetic analysis by maximum likelihood. *Mol Biol Evol*. 24(8):1586–91

Yu G, Smith DK, Zhu H, Guan Y, Lam TT-Y. 2017. ggtree: an r package for visualization and annotation of phylogenetic trees with their covariates and other associated data. *Methods in Ecology and Evolution*. 8(1):28–36
